# Molecular characteristics of primary pulmonary lymphoepithelioma-like carcinoma based on integrated genomic analyses

**DOI:** 10.1038/s41392-020-00382-6

**Published:** 2021-01-08

**Authors:** Bojiang Chen, Yu Zhang, Sisi Dai, Ping Zhou, Wenxin Luo, Zhoufeng Wang, Xuping Chen, Peng Cheng, Guoya Zheng, Jing Ren, Xiaodong Yang, Weimin Li

**Affiliations:** 1 Department of Respiratory and Critical Care Medicine, West China Hospital of Sichuan University, No. 37, Guo Xue Alley, 610041 Chengdu, Sichuan China; 2 Frontiers Science Center for Disease-related Molecular Network, West China Hospital, Sichuan University, Chengdu Sichuan, China; 3 Novogene Co., Ltd, Beijing, China; 4 Department of Pathology, West China Hospital of Sichuan University, Chengdu, Sichuan China; 5 Department of Respiratory and Critical Care Medicine, Guangyuan Central Hospital, No. 16, Jing Jia Alley, Lizhou District, Guangyuan,, 628099 Sichuan China

**Keywords:** Lung cancer, Cancer genomics

## Abstract

Primary pulmonary lymphoepithelioma-like carcinoma (pLELC) is a rare non-small cell lung cancer (NSCLC) subtype. Clinical features have been described in our previous report, but molecular characteristics remain unclear. Herein, pLELC genomic features were explored. Among 41,574 lung cancers, 128 pLELCs and 162 non-pLELC NSCLCs were enrolled. Programmed cell death ligand 1 (PD-L1) and protein 53 (p53) expression was detected in 47 surgically resected pLELC samples by immunohistochemical assays. Multiomics genomic analyses, including whole-genome sequencing (WGS), RNA whole-transcriptome sequencing (RNA-seq), and Epstein-Barr virus (EBV) integration analyses, were performed on eight frozen pLELC tissues and compared with 50 lung adenocarcinomas (LUADs) and 50 lung squamous cell carcinomas (LUSCs) from The Cancer Genome Atlas (TCGA) and another 26 EBV-positive nasopharynx cancers (EBV^+^-NPCs). Progression-free survival (PFS) and overall survival (OS) of pLELC patients were better than those of non-pLELC patients. High PD-L1 or p53 expression was associated with extended disease-free survival (DFS). pLELC had 14 frequently mutated genes (FMGs). Somatically mutated genes and enrichment of genetic lesions were found, which differed from observations in LUAD, LUSC, and EBV^+^-nasopharyngeal carcinoma (NPC). Three tumor-associated genes, zinc finger and BTB domain-containing 16 (*ZBTB16*), peroxisome proliferator activated receptor gamma (*PPARG*), and transforming growth factor beta receptor 2 (*TGFBR2*), were downregulated with copy number variation (CNV) loss. EBV was prone to integrating into intergenic and intronic regions with two upregulated miR-BamH1-A rightward transcripts (*BART*s), *BART5-3P* and *BART20-3P*. Our findings reveal that pLELC has a distinct genomic signature. Three tumor-associated genes with CNV loss and two miR-*BART*s might be involved in pLELC tumorigenesis.

## Introduction

Lymphoepithelioma-like carcinoma (LELC) is a histologically distinct tumor subtype that may invade several organs, such as the lungs, nasopharynx, and stomach.^1,2^ Primary pulmonary LELC (pLELC) is categorized into the other and unclassified non-small cell lung cancer (NSCLC) group according to the 2015 World Health Organization (WHO) classification,^3^ accounting for <1% of all NSCLC cases.^4^ In our previous investigation, the clinicopathological features of 42 pLELCs and 134 pulmonary squamous carcinomas were compared. The results revealed that, unlike pulmonary squamous carcinoma, middle-aged women and nonsmokers predominated among pLELC patients. No epidermal growth factor receptor (*EGFR*) mutation, anaplastic lymphoma kinase (*ALK*) gene rearrangement, or c-ros oncogene 1 (*ROS1*) fusion was found.^5^ Due to its low incidence and the lack of information regarding the molecular mechanism of its tumorigenesis, treatment strategies for pLELC have not been fully defined, although surgery, chemotherapy, and radiation are often used.^6^ With the rise of immunotherapies, monoclonal antibodies against programmed cell death ligand 1 (PD-L1) have attracted researchers’ attention in pLELC treatment. A potential benefit has been reported in some pLELC cases.^7^ However, the prognostic significance of PD-L1 expression in pLELC is still completely controversial^8–10^

In recent years, a few studies have reported the mutational landscape of pLELC using multiple approaches, including whole-exome sequencing, targeted deep sequencing, and single-nucleotide polymorphism (SNP) arrays.^11,12^ However, analysis at the RNA level, e.g., transcriptome sequencing, seems to be absent in relevant research. In addition, Epstein–Barr virus (EBV), which has been detected in various tumors, such as Burkitt’s lymphoma, EBV-associated gastric cancer, and nasopharyngeal carcinoma (NPC),^2^ as well as pLELC,^5^ either exists typically as an episome in cells or tends to integrate into the host genome. This mechanism facilitates the expression of viral protein and microRNAs (miRNAs) and in turn promotes the pathogenesis and progression of tumors.^13^ Our previous study suggested that the serum EBV DNA level appeared to be a predictor of progression-free survival (PFS) for pLELC patients,^5^ but the exact EBV integration loci and pathogenesis are still unknown.

To explore the molecular characteristics of pLELC with multi-genomic analyses, we extended our pLELC sample size to 128 cases, with 162 non-pLELC NSCLCs as controls. All the subjects were identified from a database of 41,574 lung cancers. Whole-genome sequencing (WGS) and RNA whole-transcriptome sequencing (RNA-seq) combined with EBV integration analysis were performed on eight fresh-frozen tissues of pLELC, and distinct molecular features were identified on comparison of lung adenocarcinoma (LUAD), lung squamous cell carcinoma (LUSC), and EBV-positive nasopharynx cancer (EBV^+^-NPC). To evaluate factors influencing survival in pLELC, PD-L1 expression was detected by immunohistochemical (IHC) analysis of surgically resected paraffin-embedded samples, as well as *TP53* mutation, which has been implicated in the regulation of PD-L1 in oncogenesis.^14,15^ The results of this study confirmed a favorable outcome of pLELC in a relatively large cohort. High expression of PD-L1 or wild-type p53 was associated with extended survival. The genome studies revealed a distinct mutational signature of pLELC different from those of LUAD, LUSC, and EBV^+^-NPC. Three tumor-associated genes were identified, which were clearly downregulated with simultaneous copy number variation (CNV) loss. EBV tends to integrate into intergenic and intronic regions in pLELC with two miR-*Bam*H1-A rightward transcripts (*BART*s), *BART5-3P* and *BART20-3P*, which are upregulated. These findings provide novel insights into the molecular mechanisms of pLELC.

## Results

### Clinical features of pLELC and non-pLELC NSCLC patients

**Fig. 1:**
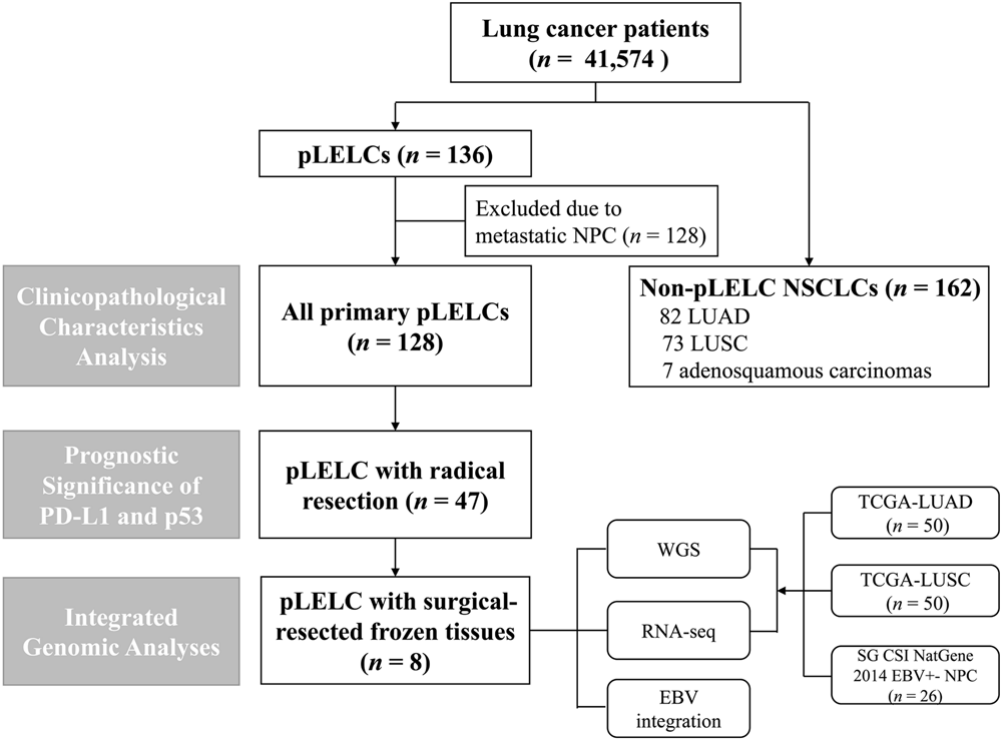
Study profile. This flow chart depicts the enrollment and exclusion of all subjects. pLELC pulmonary lymphoepithelioma-like carcinoma, NPC nasopharyngeal carcinoma, LUAD lung adenocarcinoma LUSC lung squamous cell carcinoma, WGS whole-genome sequencing, RNA-seq RNA whole-transcriptome sequencing. The eighth patient (P8) numbered in the multi-genomic sequencing was excluded from the whole data analysis process because of metastatic NPC even though the sample had already been sequenced. Therefore, P9 was included in the integrated genomic analyses but P8 was not

The workflow of this study is shown in Fig. 1. The clinicopathological characteristics of the 128 primary pLELC and 162 non-pLELC NSCLC cases are summarized in Table 1. The mean age of the pLELC patients was substantially younger than that of the controls, and a female predominance was noted. Smoking was much less common among pLELC patients. For tumor, node, metastasis (TNM) and clinical staging, pLELC patients seemed to be more prone to distant lymph node invasion and distant metastasis when diagnosed; therefore, fewer pLELC patients had stage I and II disease compared to NSCLC patients. These data suggested that pLELC may be a special type of NSCLC.

**Table 1:** Clinical characteristics of pLELCs and non-pLELC NSCLCs

Variables	pLELCs	Non-pLELC NSCLCs	*P*
	*n* (%)	*n* (%)	
Age (mean ± SD, years)	54.6 ± 9.5	60.2 ± 10.1	<0.0001*
Gender			
Female	76 (59.4)	56 (34.6)	<0.0001*
Male	52 (40.6)	106 (65.4)	
Smoking			
Yes	26 (20.3)	105 (64.8)	<0.0001*
No	102 (79.7)	57 (35.2)	
Family history of cancer			
Yes	18 (14.1)	21 (13.0)	0.785
No	110 (85.9)	141 (87.0)	
TNM stage (TNM data were available for only 104 pulmonary LELC cases)			
T			
1	28 (26.9)	17 (10.5)	<0.0001*
2	23 (22.1)		76 (46.9)
3	22 (21.2)		44 (27.2)
4	31 (29.8)		25 (15.4)
N			
0	41 (39.4)	81 (50.0)	0.001*
1	13 (12.5)		31 (19.1)
2	37 (35.6)		47 (29.0)
3	13 (12.5)		3 (1.9)
0 + 1/2 + 3	54/50 (51.9/48.1)	112/50 (69.1/30.9)	0.005*
M			
0	80 (76.9)	152 (93.8)	<0.0001*
1	24 (23.1)		10 (6.2)
Clinical stage			
I	25 (24.0)	52 (32.1)	0.001*
II	18 (17.3)	39 (24.1)	
III	37 (35.6)	61 (37.6)	
IV	24 (23.1)	10 (6.2)	
I + II/III + IV	43/61 (41.3/58.7)	91/71 (56.2/43.8)	0.018*

*P < 0.05 with significant difference

**Fig. 2:**
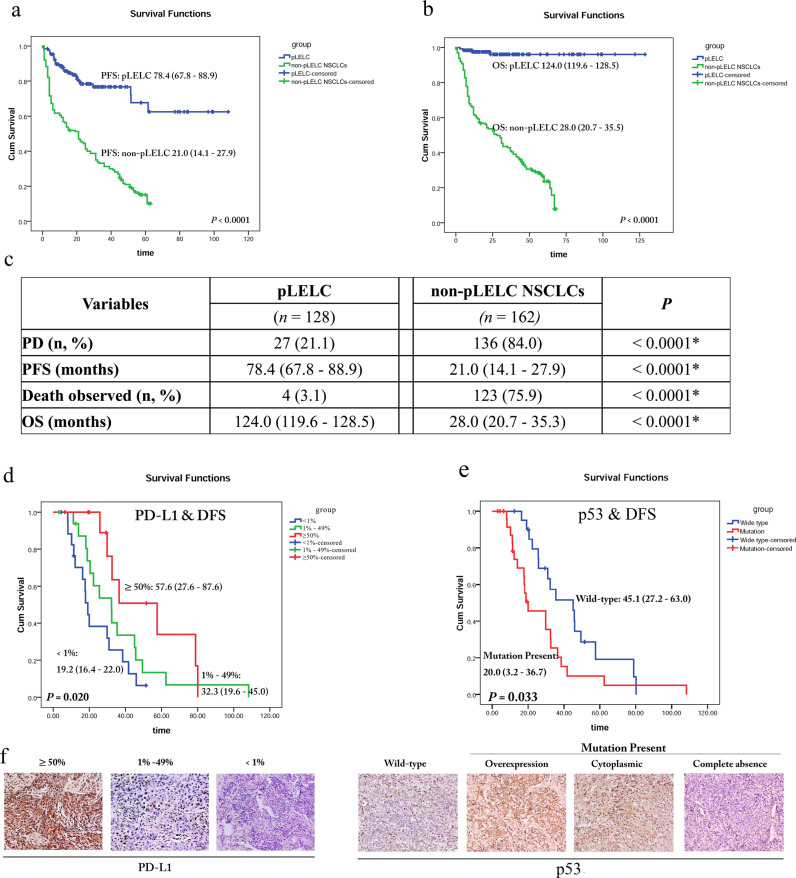
Prognosis of pLELC by Kaplan–Meier survival analyses with log-rank test. **a** The PFS probability of pLELC patients compared with non-pLELC NSCLC patients (78.4 months vs. 21.0 months, *P*<0.0001). **b** The OS probability of pLELC patients compared with non-pLELC NSCLC patients (124.0 months vs. 28.0 months, *P*<0.0001). **c** Information in detail. *P<0.05 with a significant difference. Both PFS and OS are estimated by the mean PFS value due to the high censor rate. **d** The DFS probability of pLELC patients with different PD-L1 expression levels in tumor cells (median DFS: 19.2 vs. 32.3 vs. 57.6 months for <1%, 1–49%, and ≥50% PD-L1 expression, respectively, *P*=0.020). **e** The DFS probability of pLELC patients with mutation-type or normal p53 expression (median DFS: 20.0 vs. 45.1 months respectively, *P*=0.033). **f** Representative images of PD-L1 and p53 expression by IHC (magnification ×400). *P*<0.05 indicates a significant difference

The median follow-up time was 25.6 months (2.87–128.6 months). As shown in Fig. 2a, c, disease progression was observed more infrequently in pLELC patients than in controls. Consistently, the median PFS of pLELC patients, estimated by the mean PFS value due to the high censor rate, was 78.4 months, which was substantially more favorable than that of non-pLELC patients.

In the overall survival (OS) analyses (Fig. 2b, c), the number of patients who had died since diagnosis was 4 vs. 123 in the pLELC and non-pLELC NSCLC cohorts, respectively. The median OS of the pLELC patients, also estimated by the mean OS value because of the high censor rate, was 124.0 months, which was also remarkably better than that of the controls.

### Prognostic significance of PD-L1 and p53 expression in pLELC

Among the 128 pLELC patients, 47 patients who underwent radical surgery were enrolled for expression detection and prognostic significance analyses of nuclear p53 and membranous/cytoplasmic PD-L1. Considering that no deaths occurred in the pLELC cohort until the last follow-up exam and only ten patients showed disease progression, disease-free survival (DFS) was analyzed rather than OS. As shown in Table 2, the mean age of this cohort was 54.4 years. Then the patients were divided into those aged <50 years and those aged >50 years for DFS association analysis, but no significance was found. Negative correlations were also found for gender, smoking, family history of cancer, and clinical stage.

**Table 2:** Prognostic significance of PD-L1 and p53 expression levels, along with clinical variables, in the radically resected pLELC cohort (*n* = 47)

Variables	*n* (%)	Median DFS/months	*P*
Age (mean ± SD, years)		54.4 ± 8.8	
Age			
<50 years	13 (27.6)	30.9 (15.6–46.2)	0.665
≥50 years	34 (72.4)	32.3 (24.2–40.4)	
Gender			
Female	29 (61.7)	32.3 (29.1–35.5)	0.666
Male	18 (38.3)	25.6 (11.9–39.3)	
Smoking			
Yes	12 (25.5)	25.6 (5.2–46.0)	0.466
No	35 (74.5)	30.9 (27.4–34.4)	
Family history of cancer			
Yes	6 (12.8)	45.7 (30.2–61.2)	0.242
No	41 (87.2)	29.8 (19.7–40.0)	
Clinical stage			
I	19 (40.4)	29.9 (11.4–48.3)	0.185
II		7 (14.9)	18.1 (1.0–35.5)
IIIA		21 (44.7)	35.4 (15.3–55.5)
PD-L1 in tumor cells			
<1%	18 (38.3)	19.2 (16.4–22.0)	0.020*
1–49%		17 (36.2)	32.3 (19.6–45.0)
≥50%		12 (25.5)	57.6 (27.6–87.6)
p53			
Wild type	21 (44.7)	45.1 (27.2–63.0)	0.033*
Mutation type		26 (55.3)	20.0 (3.2–36.7)

*P < 0.05 with significant difference

For the PD-L1 and p53 expression investigation, 61.7% (29/47) of the samples were positive for PD-L1, including 12 strong and 17 moderate cases. Notably, patients with strong expression of PD-L1 demonstrated the longest median DFS among the three groups (Fig. 2d, f). Moreover, mutation-type p53 expression was observed in more than half of the cohort (26/47), and this group showed shorter DFS than the group with normal p53 expression (median DFS 20.0 months vs. 45.1 months; Fig. 2e, f). As abnormal expression of p53 is a strong predictor of an underlying *TP53* mutation, the results indicated that *TP53* mutation status is correlated with the prognosis of pLELC.

Taken together, the prognosis data above suggest that pLELC might be a particular type of NSCLC, which is usually at an advanced stage at the time of diagnosis but has a favorable prognosis, especially with high expression of PD-L1 and wild-type p53. However, molecular features of pLELC remain unclear, which prompted us to carry out subsequent multiomics studies.

### Integrated genomic analyses of pLELC

#### Mutation burden and mutation spectrum of pLELC

**Fig. 3:**
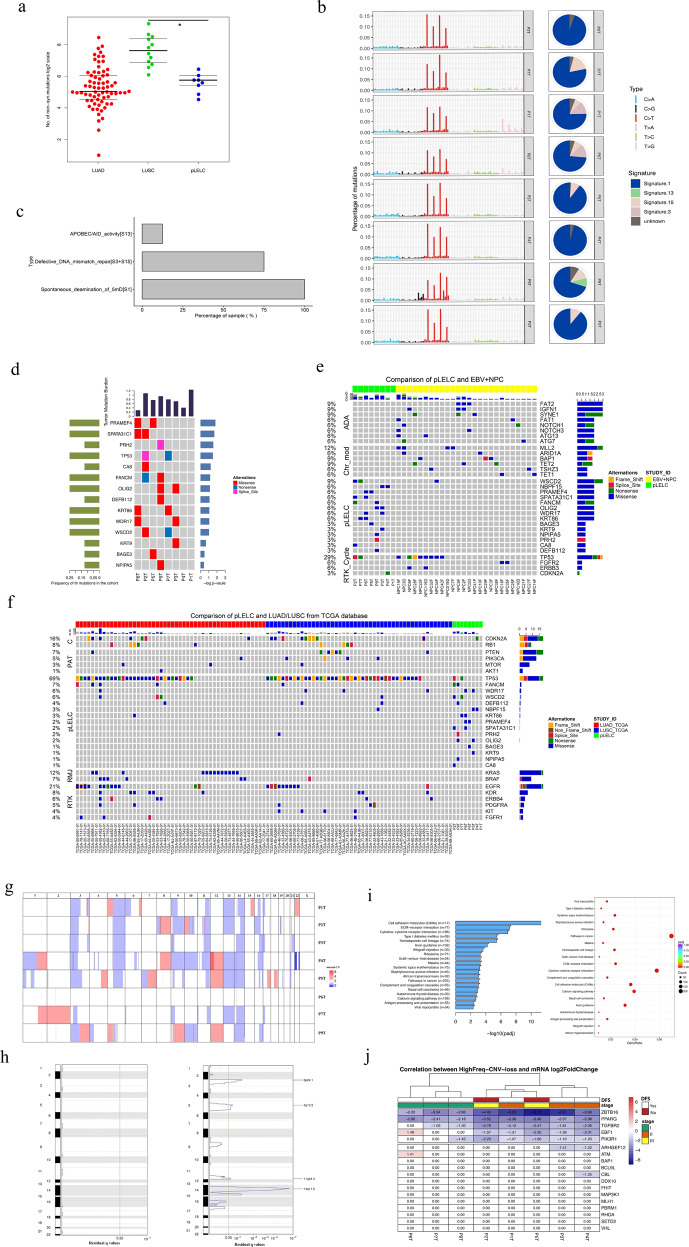
Mutation spectrum and CNV landscape of pLELC with enrichment analysis. **a** Nonsynonymous mutation counts in LUAD (red), LUSC (green), and pLELC (blue); the *y*-axis represents the log2 count. **b** Mutation signature analysis in patients with pLELC. Each subgraph shows a single pLELC sample, and the bar displays different base alteration types, with the pie chart depicting the proportions of different signature types. **c** Percentages of samples harboring different mutation signatures. **d** Mutation landscapes of eight pLELC samples. The red, blue, and pink represent missense, nonsynonymous, and shear site mutations, respectively. The bar on the left shows the mutation frequency in the population. **e** Comparison of genomic mutation profiles between pLELC and NPC. **f** Comparison of genomic mutation profiles between pLELC, LUAD, and LUSC. The bars at the top of the chart represent different tumor types. The color blocks represent different types of base alterations, with the mutation frequency of the population presented on the left. **g** Overview of the pLELC CNV landscape. Each row represents one sample; the red and blue squares represent gains and losses, respectively, and the *x*-axis represents the chromosome number of the whole human genome. **h** High-frequency CNVs of segments in pLELC. CNV gain is shown on the left, and CNV loss is shown on the right. The purple line on the right represents a wide peak limit. **i** Pathway enrichment analysis. Left: the *x*-axis represents −log2 values (adjusted *P* values), and the *y*-axis represents the different signaling pathways and the number of genes involved. Right: the *x*-axis represents the gene ratio in each pathway, and the red circle denotes a low adjusted *P* value. **j** Integrated analyses of the transcriptome combined with genome CNV. Each column represents a sample, and each row represents the gene with high-frequency CNV loss. At the top of the diagram are annotations of the sample clinical information, representing the DFS time, as well as the clinical staging. The figure shows the mRNA expression of this gene in the cancer sample relative to the control log2-fold change. *ZBTB16* and *PPARG* were lost and downregulated in 100% (8/8) of patients with pLELC; the mRNA log2-fold changes in *ZBTB16* and *PPARG* were −2.20 to −6.15 and −2.37 to −3.46, respectively. *TGFBR2* was lost and downregulated in 87.5% (7/8) of patients, with a −1.09 to −2.78 log2-fold change in mRNA. NPC nasopharyngeal carcinoma, LUAD lung adenocarcinoma, LUSC lung squamous cell carcinoma

To further understand the molecular characteristics of pLELC and explore its mechanisms of tumorigenesis, WGS was performed on eight tumor specimens, the clinicopathological characteristics of which are listed in Supplementary Table 1. Because pLELC is extremely similar to LUSC in histopathology, complicating the differential diagnosis, their genomes were compared first and then together with that of LUAD. The overall nonsynonymous mutation rate of pLELC was significantly lower than that of LUSC (*P* = 0.00002; unpaired *t* test; Fig. 3a). Strong enrichment of the C>T transition was found in all eight pLELC samples (Fig. 3b), followed by enrichment of T>G transitions (P1 and P6). A combination of non-negative matrix factorization clustering and correlations with the 30 curated mutational signatures defined by the Catalogue of Somatic Mutations in Cancer (COSMIC) database^16,17^ revealed four dominant signatures (Fig. 3b). The predominant signatures were the deamination process of 5-methyl-cytosine (signature 1), followed by defective DNA mismatch repair (signatures 3+15) and the APOBEC/AID signature (signature 13) (Fig. 3b, c).

Chromothripsis is a phenomenon where hundreds of chromosomal rearrangements are generated via a single catastrophic event. In this study, chromothripsis was found in 12.5% (1/8) of pLELC samples (Supplementary Fig. 1a–c). Compared with the other pLELC patients, the patient with chromothripsis had considerably shorter DFS (Supplementary Fig. 1d). However, whether chromothripsis is prevalent in pLELC requires further investigation in larger cohorts.

#### Somatic mutations of pLELC

A total of 14 frequently mutated genes (FMGs) with abnormally high levels were found in the pLELC group (Fig. 3d). To explore the unique driver genes in pLELC, typical somatic mutation profiles were compared with those of other lung cancer subtypes (50 LUADs and 50 LUSCs in The Cancer Genome Atlas (TCGA)) and 26 EBV^+^-NPC cases.^18^ Although carcinogenesis of both pLELC and NPC is related to EBV infection, somatic mutations of pLELC were found to be dramatically different from those of EBV^+^-NPC (Fig. 3e). Simultaneously, the typical driver genes reported in LUAD and LUSC were scarcely detected in pLELC except for *TP53* and *CDKN2A* (Fig. 3f). Beyond the different mutation spectra, the number of FMGs in pLELC was much lower than those in LUAD, LUSC, and EBV^+^-NPC. These data suggest a unique etiological mechanism of pLELC.

Then somatic copy number alterations were profiled using the Control-FREEC tool^19^ and GISTIC software with a cutoff of 0.05. CNV loss was widely observed in pLELCs (Fig. 3g), and the most significant CNV loss occurred in 3p24.1, 5q12.3, 11q24.3, and 14q11.2 (Fig. 3h). The results show that CNV loss rather than single-nucleotide variations may underlie the carcinogenesis of pLELC.

#### Kyoto Encyclopedia of Genes and Genomes (KEGG) pathway enrichment with differentially expressed unigenes (DEGs)

DEG analysis was performed for each patient by comparing tumors and adjacent normal lung tissues using Cufflinks and RSEM, and KEGG pathway enrichment was analyzed by EdgeR^20,21^ with a corrected *P* < 0.05 (multiple-hypothesis correction by the Benjamini–Hochberg false discovery rate method) indicating significant enrichment. A total of 458 DEGs were enriched with 20 significant signaling pathways, ranging from 7.6 to 48.2% (Fig. 3i). The four mainly enriched KEGG pathways were further mapped into 74, 32, 19, and 11 subpathways (Supplementary Fig. 2).

#### Integrated genomic and transcriptome analyses

Using integrated genomic and transcriptome analyses, copy number and expression decreases in five tumor-associated genes (zinc finger and BTB domain-containing 16 (*ZBTB16*), peroxisome proliferator activated receptor gamma (*PPARG*), transforming growth factor beta receptor 2 (*TGFBR2*), early B cell factor 1 (*EBF1*), and phosphoinositide-3-kinase, regulatory subunit 1 (alpha) (*PIK3R1*)) were detected in pLELC, especially *ZBTB16*, *PPARG*, and *TGFBR2* (Fig. 3j). Patients in the more advanced stage also seemed to exhibit a more distinct reduction in tumor-associated genes, which might be a potential pathogenesis of pLELC (Fig. 3j).

#### EBV integration analysis

**Fig. 4:**
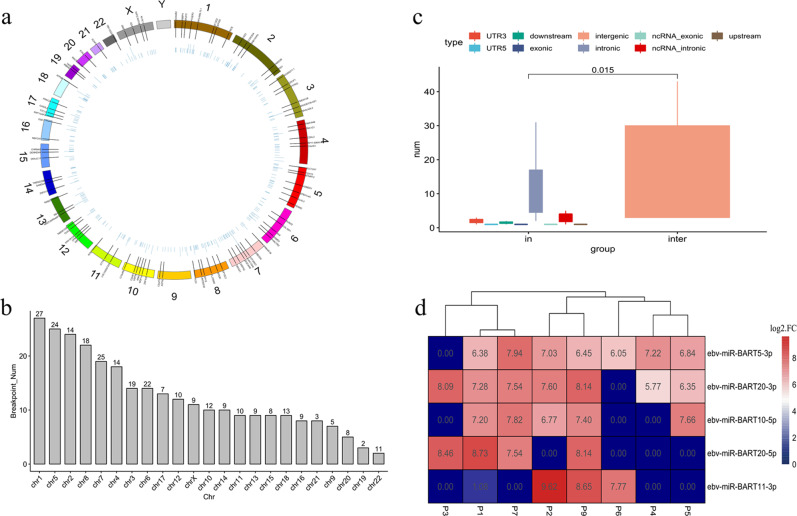
EBV integration and EBV-encoded miRNA analysis. **a** Landscape of EBV integration breakpoints over the whole genome. Chromosomes are numbered and represented by a range of colors. **b** The distribution of EBV integration breakpoints among chromosomes. The *x*-axis is the chromosome number, and the *y*-axis represents the number of integration breakpoints. **c** The preference of EBV integration loci. The integration positions were classified into intragenic and intergenic regions. Intergenic regions tend to be the most favorable integration loci for EBV, followed by intronic regions (*P* = 0.015). **d** EBV miRNA cluster analysis. The number of each block (*N*) is calculated using the equation: *N* = log_2_(mRNA expression level of a certain gene in tumor tissue/mRNA expression level of a certain gene in control tissue). Blue represents downregulated mRNA, and red represents upregulated mRNA

The WGS data were aligned with EBV reference genomes to perform comprehensive profiling of EBV integration. The average percentage of EBV sequences in the WGS data was 0.14% (0.10–0.25%), and the coverage depth was 220.69× on average (40–1005×). Moreover, EBV genome contents in tumor tissues were found to be greater than those in normal samples, with a larger percentage and a greater depth of reads mapped on the EBV genome (Supplementary Fig. 3). A total of 288 EBV integration breakpoints were identified, and 268 breakpoint sites were found in five patients (P2, P3, P4, P5, and P7) (Supplementary Material 1, available online only). These breakpoint sites were dispersed over 23 human chromosomes, except for the Y chromosome (Fig. 4a, b). Eight pairs of integration breakpoints were found to be ubiquitous, among others, by analyzing the breakpoint distribution in each patient (Supplementary Fig. 4 and Supplementary Table 2). Through gene annotation of the integration sites, EBV was found to have a strong tendency to integrate into intergenic rather than intragenic regions, predominantly within intronic regions (Fig. 4c). In addition, 19 (6.6%) integration sites were localized within noncoding RNA introns, two of which were localized within the introns of HNF1A-AS1 and LINC00673 in two patients (P3 and P5, Supplementary Fig. 5). EBV integration hotspots seemed to be fragile genomic regions, which were vulnerable to DNA damage. This feature increased the likelihood of EBV DNA insertion through microhomology-mediated DNA repair, as shown in Supplementary Fig. 5.

#### Expression profiles of EBV-encoded miRNAs in pLELC

To explore the EBV-encoded miRNA expression patterns in pLELC, small RNA sequencing was performed on eight pLELC patients. In each sample, approximately 0.4–4.1 (80.2–85.5%) million reads mapped to the EBV genome were obtained. All of the reads (100%) mapped to the EBV genome were known EBV-encoded miRNAs (*Bam*H1 fragment H rightward open reading frame 1, miR-*BHRF1*; and miR-*BART*), indicating that miRNAs were significant components of EBV-derived small RNAs in pLELC (Supplementary Fig. 6). The individual miR-*BART* frequency ranged from 9.1 to 34.1% (Supplementary Table 3). In addition, among 43 identified miRNAs in EBV, a total of 31 (72.1%) differentially expressed miR-*BART*s were detected compared with adjacent normal lung tissues (Supplementary Material 2, available online only). Two *BART*s (*BART5-3P* and *BART20-3P*) were frequently (7/8, 87.5%) upregulated, and the other three (*BART10-5P*, *BART20-5P*, and *BART11-3P*) were relatively less changed (Fig. 4d and Supplementary Table 4). Additionally, the six patients with both *BART5-3P* and *BART20-3P* upregulation had low p53 and PD-L1 expression and displayed a relatively poor prognosis.

## Discussion

pLELC is a rare subtype of NSCLC characterized by EBV infection without a unique pathological presentation. In the present study, we assessed PD-L1 and p53 expression in 47 pLELCs and performed an integrated genomic analysis of 8 pLELCs using WGS and RNA-seq. Our work confirmed previous results reported in other studies indicating that females and nonsmokers are predominantly affected by pLELC and that patients with pLELCs usually show high PD-L1 expression.^10^ Furthermore, the mutation spectrum of pLELC was found to be distinct from those of other subtypes of NSCLC and EBV^+^-NPC, with unique FMGs and widespread CNV loss. As EBV infection plays an important role in the tumorigenesis of pLELC, we studied EBV integration loci as well as EBV-encoded miRNA expression in host cells. In summary, our work shed lights on the distinct genetic features of pLELC and the mechanism underlying its pathological characteristics.

In this study, 61.7% (29/47) of samples were positive for PD-L1 by IHC 28-8. This result is basically similar to previous findings indicating that 66–76% of pLELCs are positive for PD-L1-stained tumor cells.^22^ High expression of PD-L1 has been suggested to be a biomarker for survival prediction and optional immunotherapy, while *TP53* mutation and membranous PD-L1 expression levels were highly correlated in NSCLC.^22^ Here we explored the prognostic significance of PD-L1 expression and *TP53* mutation status in pLELC and found that pLELC patients with high PD-L1 expression tend to have longer DFS, which is consistent with previous studies.^22^ In contrast, patients with wild-type p53 expression showed a better prognosis in our cohort, although the interpretation of p53 IHC varies across studies. Further studies with subjects from multiple centers are needed to confirm the value of PD-L1 and p53 in pLELC.

In our previous report, in a susceptible population, computed tomography (CT) appearances and prognosis of pLELC were all markedly different from those of LUSC.^5^ We extended the cohorts of pLELC and controls for systematic comparison at a molecular level. First, the genomic mutation profile of pLELC was compared with those of LUSC and LUAD. Genes with high mutation frequencies in thoracic malignancies were barely observed in pLELC. The lack of powerful mutation drivers might render pLELC tumor cells less aggressive and may lead to increased survival in pLELC patients. Moreover, as an EBV-inter-related neoplasm, pLELC is very closely related to EBV^+^-NPC in histopathological characteristics, complicating the differential diagnosis in clinical practice. Therefore, genomic mutations of pLELC and EBV^+^-NPC were compared. However, no similarity was found. Lin et al. demonstrated that mutations of chromosomal-modifying genes and the *ERBB*-*PIK3CA* signaling pathway were important in NPC, directly affecting epigenetic modification and promoting invasion.^18^ These abnormalities were widely observed in other malignant tumors.^23,24^ However, in the present study, no mutations of chromosome-modification pathways were detected; additionally, no mutations of *RTK* cycle pathway-related genes were tested, which may explain why pLELC is less aggressive than NPC.

In the integrated genomic and transcriptome analyses, the tumor-associated genes *ZBTB16*, *PPARG*, and *TGFBR2* were found to be significantly downregulated with simultaneous CNV loss. *ZBTB16* is also known as promyelocytic leukemia zinc-finger protein (*PLZF*). As a transcriptional repressor, *ZBTB16* has an important role in cell death, differentiation, and tumor progression.^25^ Downregulation of *ZBTB16* was observed in pancreatic cancer and prostate cancer,^26,27^ and the high expression of *ZBTB16* was associated with long-term survival in cancer patients.^28,29^ In our study, *ZBTB16* was reduced, and this reduction might be one of the possible factors associated with pLELC tumorigenesis.

*PPARG* belongs to the nuclear receptor superfamily of *PPAR*s. Several studies have concentrated on the key role of *PPARG* in cancers with inflammatory regulation.^30^ *PPARG* was also found to be involved in the regulation of epithelial–mesenchymal transition (EMT).^31^ However, activated *PPARG* could reverse the mesenchymal–epithelial phenotype and inhibit EMT.^32^ In hepatocellular carcinoma and breast cancer, *PPARG* showed antitumorigenic effects.^30^ In contrast, *PPARG* has been reported as an immunomodulator. High expression of *PPARG* impairs CD8+ T cell infiltration in bladder cancer and confers resistance to immunotherapies, while knockdown/inhibition of *PPARG* increases cytokine expression and revives immunosurveillance.^33^ Therefore, we speculated that the loss of *PPARG* in pLELC might improve immunosurveillance, leading to a good prognosis in pLELC cases.

*TGF*-*β* is the prototype of the *TGF*-*β* family. During tumor progression, TGF-β becomes a stimulating molecule that promotes the growth, invasion, and metastasis of tumor cells by inducing immune escape.^34^ Furthermore, TGF-β acts as an immunosuppressor by affecting the regulation of T cells.^35^ A study on urothelial cancer showed that TGF-β expression was increased in PD-L1 nonresponders and that simultaneously blocking TGF-β and PD-L1 could facilitate T cell infiltration and provoke antitumor immunity and tumor regression.^36^ Accordingly, *TGF*-β inhibition in pLELC patients was inferred to induce an immune response to promote survival.

Taken together, the loss of *ZBTB16* can be hypothesized to improve pLELC tumorigenesis, while reductions in *PPARG* and *TGFBR2* may promote antitumor immunity, leading to a favorable prognosis.

Similar to previous studies by Hong et al. and Xie et al.,^11,12,37^ *TP53* mutation was also detected in our study. Except for *TP53*, no other common FMGs were observed within these studies, but a variety of similar CNVs were identified at both the segment and gene levels. We assume that the reasons included the different panels used in NGS and variable sample sizes. In addition, Signature 13 detected in the present study is essentially similar to Signature 2 reported by Hong et al.^11^ because both of their biological functions are related to overactivity of APOBEC/AID. The number discrepancy is due to different sig database versions.

EBV infection has been linked to various human malignancies.^2^ The EBV genome, which typically exists as an episome or is integrated within the host genome through the microhomology of integration, can promote the pathogenesis and progression of tumors.^13^ In this study, all pLELC tumor tissues were positive in the EBV-encoded RNA (EBER) test. In addition, a total of 288 EBV integration breakpoints were determined by aligning the WGS data to the EBV reference genome. Two breakpoint sites were localized within the introns of HNF1A-AS1 and LINC00673 in pLELC-P5 and pLELC-P3, respectively. Similar integration was also found in EBV-associated colon cancer and regulated the progression of malignant cells.^38^ Unfortunately, no tumor suppressor or oncogenes were discovered through inspection of the upstream (20 kb) and downstream (200 kb) breakpoints. In NPC, EBV is integrated into introns of the inflammation-related genes *PARK2*, tumor necrosis factor alpha-induced protein 3 (*TNFAIP3*), and cyclin-dependent kinase 15 (*CDK15*), which regulate the nuclear factor-κB pathway.^13^ However, this behavior was not observed in pLELC. Moreover, EBV was reported to integrate near common fragile sites, avoiding short interspersed nuclear elements in NPC.^13^ In contrast, intergenic regions were the favored integration site for EBV in pLELC in our study. These fragile regions are vulnerable to DNA damage, which increases the likelihood of EBV DNA insertion into host genomes. The functional impact of such integration needs to be investigated in future studies.

EBV encodes two groups of miRNAs, *BHRF1* and *BART*. A total of 48 mature miRNAs, including 4 miR-*BHRF1*s and 44 miR-*BART*s, are generated from 25 miRNA precursors.^39^ In NPC, high expression of miR-*BART*s contributes to cancer development by targeting various cellular and viral genes.^40^ Moreover, *BART3* and *BART9* were observed to be closely associated with cell growth and proliferation,^41^ while *BART5* and *BART10* played crucial roles in cell apoptosis.^42^ In addition, *BART1* induced tumor metastasis and recurrence in NPC by directly targeting the dominant tumor suppressor, phosphatase and tensin homolog (*PTEN*), and consequently activating *PTEN*-dependent pathways and inducing EMT.^43^ *BART5-3P* and *BART10-5P* were also reported to be upregulated in NPC and to participate in the regulation of cell apoptosis and proliferation.^44^ In this study, *BART5-3P* and *BART20-3* were found to be upregulated in pLELC, but *BART1* was not identified. The expression of these EBV-miRNAs was inferred to contribute to pLELC tumorigenesis and development.

In addition, *BART5-3P* has been reported to inhibit p53 protein expression.^45^ In the current study, cases with *BART5-3P* overexpression were found to have low expression of p53 and PD-L1 and a poor prognosis. Combined with report indicating that PD-L1 was correlated with p53 in NSCLC and oral squamous cell carcinoma,^14,15^ EBV insertion can be assumed to promote the expression of *BART5-3P*, consequently inhibiting p53 and PD-L1. However, the effects of *BART20-3* on tumors have not been reported. Investigating the function of these miR-*BART*s in pLELC and validating their regulatory role and downstream molecular mechanism may be of great significance in the future.

In conclusion, this study identified a distinct mutational signature in pLELC. Three tumor-associated genes were found to be downregulated with simultaneous CNV losses. In addition, this study provided an unbiased large-scale genome-wide analysis of the EBV integration landscape in pLELC. The potential targets of miRNA-derived EBV provided another clue to explore the pathogenesis of EBV in pLELC. Based on these findings, *ZBTB16* with missing CNVs and EBV-encoded *BART5-3P* expression might be key in the tumorigenesis of pLELC, whereas the reductions in *PPARG* and *TGFBR2* and the low mutation rate partially explained the favorable prognosis of pLELC.

Certainly, several limitations exist in this study. The most obvious weakness is the small sample size. Due to the rare incidence, only 128 patients had primary pLELC among the 41,574 cases. Therefore, this cohort is actually relatively large. Multicenter clinical research might be required to enroll more subjects. For genomic sequencing, only fresh-frozen tissues qualified for the analysis, and we recruited eight patients over the past few years. Although cases were limited, the comparatively deep analysis partly compensated for these shortcomings. Additionally, this is an observational investigation without interventional exploration. We will conduct further research on the mechanism of pLELC in the future.

## Materials and methods

### Study population

A total of 41,574 cases of lung cancer were enrolled in the Newly Diagnosed Lung Cancer Database of West China Hospital of Sichuan University, China between January 2008 and December 2019. A total of 136 patients were diagnosed with pLELC. However, eight patients were excluded due to metastatic NPC, and the remaining 128 primary pLELC patients, accounting for 0.31% of all lung cancer cases, were recruited. Another 162 non-pLELC NSCLC patients who were hospitalized on the same day and treated by the same doctor as the pLELC patients were qualified as controls. These enrollment criteria minimized the effects of doctors’ clinical preferences as much as possible. The control group included 82 LUADs, 73 LUSCs, and 7 adenosquamous carcinoma cases.

All patients were pathologically diagnosed according to the WHO classification criteria,^3^ and all of the pLELCs were undifferentiated carcinomas with positive EBER staining. The TNM stage classification was based on the Eighth Edition of International Association for the Study of Lung Cancer International Staging Project.^46^ Tumor tissues from 47 radical operation patients among the 128 pLELCs were analyzed using IHC to detect the expression and clinical significance of PD-L1 and p53.

Surgically resected frozen samples, including both tumor tissues and adjacent normal lung tissues, of eight pLELC patients were subjected to WGS, RNA-seq, and EBV integration analyses. Notably, the eighth patient (P8) numbered in the multigenome sequencing was excluded from the whole data analysis process because of metastatic NPC even though the sample had been sequenced. Therefore, patient nine (P9) was included in the integrated genomic analyses instead of P8. Ethical approval was obtained from the Institutional Review Board at our center, and all patients provided written informed consent for the investigation.^47^

### Clinicopathological information and follow-up parameters

Clinical data, including demographic information (sex, age, smoking status, and family history of cancer), clinical staging, and pathology reports, were obtained from the electronic medical records retrospectively, whereas the prognostic data for OS, PFS, and DFS were received by telephone interview and systemic assessments of clinical examination and CT scans according to the Response Evaluation Criteria in Solid Tumors 1.1.^48^ The time from diagnosis until death resulting from any cause was calculated as OS. In the whole cohort of 128 pLELC patients and 162 non-pLELC NSCLC patients, the time from diagnosis to clinical or radiological progression or death was defined as PFS. However, for 47 pLELC cases receiving radical resection with PD-L1 and p53 detection, only 10 patients showed disease progression. Therefore, DFS was utilized and measured from the date of diagnosis to locoregional or distant recurrence, second primary malignancy, or death, whichever occurred first.^49^ Patients with no evidence of events or no follow-up information were documented as censored at the date of last contact on January 4, 2020.^48^

### IHC assay, WGS, and RNA-seq

The steps for the IHC assay, WGS, and RNA-seq are provided in Supplementary Materials and Methods.

### Significant mutations and oncogene/tumor-suppressor gene (TSG) analysis

MuSiC^50^ was used to identify FMGs in pLELC with a convolution test (*P* < 0.2). Somatic copy number alterations were analyzed using the GISTIC software with residual *q* values of 0.25 to identify wide peak limits. In addition, the COSMIC cancer gene census database was used to extract oncogenes/TSGs with tier 1 evidence. Somatic signature analyses were performed according to a previously described approach.^16^ The nonsynonymous mutation rates of pLELC were compared with those of other lung cancer subtypes, including 50 LUADs and 50 LUSCs from TCGA and 26 EBV^+^-NPCs obtained from another group.

### RNA quantification, DEG screening, and pathway enrichment analysis

Transcripts were quantified using Cuffdiff, and the differential expression of transcripts between different groups was calculated and analyzed using the edgeR software. Records with a *P* value <0.05 were retained for differential screening. The clusterProfiler was used to select KEGG pathways and to analyze the pathway enrichment of DEGs.^20^

### Functional annotation and filtration

ANNOVAR was performed to annotate the variant call format. Consensus Coding Sequence, RefSeq, Ensembl, and UCSC were used to determine amino acid variations. The annotation content contained variant positions, variant types, conservative predictions, etc. from the dbSNP, COSMIC, 1000g2015aug_all, and Exome Aggregation Consortium databases. ALL databases were also used to obtain the population frequencies of mutations.

### EBV-encoded miRNA detection

Details of genome alignment, host integration of EBV, and variant calling are provided in Supplementary Materials and Methods. Bowtie was used to match the length-screened sRNAs to the EBV reference sequence. Whether the reads mapped to the EBV genome were known EBV-encoded miRNAs was determined based on reports.^40,51^

### Statistical analysis

The chi-square test was utilized to compare the clinical characteristic distribution between pLELCs and non-pLELC NSCLCs for categorical factors (Fisher’s exact tests when necessary), and Student’s *t* test was used for continuous variables. Kaplan–Meier method was employed to estimate the OS, PFS, and DFS with the log-rank test for comparison. Differences in the clinical data were considered statistically significant when the *P* value was <0.05 in SPSS version 20.0 (SPSS, Inc., USA). The integrated genomic analyses were conducted as reports, and methods are clarified in the corresponding sections with references marked.

## Supplementary information

Supplementary materials

Supplementary material 1, available online only

Supplementary material 2, available online only

## Data Availability

Raw data of this project have been uploaded to the https://bigd.big.ac.cn/gsa and the code of our data was HRA000291. The online version of this article contains supplementary materials, which is available to authorized users.

## References

[1] Chaparro Mirete M, Lopez-Lopez V, Robles Campos R (2020). Lymphoepithelioma-like carcinoma of the duodenum: a very infrequent tumor. Rev. Esp. Enferm. Dig..

[2] Ayee R, Ofori MEO, Wright E, Quaye O (2020). Epstein Barr virus associated lymphomas and epithelia cancers in humans. J. Cancer.

[3] Travis WD (2015). The 2015 World Health Organization Classification of Lung Tumors: impact of genetic, clinical and radiologic advances since the 2004 classification. J. Thorac. Oncol..

[4] Qin Y (2019). Clinical features and prognosis of pulmonary lymphoepithelioma-like carcinoma: summary of eighty-five cases. Clin. Lung Cancer.

[5] Chen B (2019). Primary pulmonary lymphoepithelioma-like carcinoma: a rare type of lung cancer with a favorable outcome in comparison to squamous carcinoma. Respir. Res..

[6] Fan Y, Li C, Qin J, Lu H (2020). Primary pulmonary lymphoepithelioma-like carcinoma. Med. Oncol..

[7] Qiu ZX, Zhou P, Wang K (2019). Primary pulmonary lymphoepithelioma-like carcinoma response favorably to nivolumab: a case report. Onco Targets Ther..

[8] Fang W (2015). PD-L1 is remarkably over-expressed in EBV-associated pulmonary lymphoepithelioma-like carcinoma and related to poor disease-free survival. Oncotarget.

[9] Chang YL (2015). PD-L1 is highly expressed in lung lymphoepithelioma-like carcinoma: a potential rationale for immunotherapy. Lung Cancer.

[10] Hopkins, A. C. et al. T cell receptor repertoire features associated with survival in immunotherapy-treated pancreatic ductal adenocarcinoma. *JCI Insight* **3**, e122092 (2018). 10.1172/jci.insight.122092 PMC6124515 29997287

[11] Hong S (2019). The genomic landscape of Epstein-Barr virus-associated pulmonary lymphoepithelioma-like carcinoma. Nat. Commun..

[12] Chau, S. L. et al. Distinct molecular landscape of Epstein-Barr virus associated pulmonary lymphoepithelioma-like carcinoma revealed by genomic sequencing. *Cancers* **12**, 2065 (2020). 10.3390/cancers12082065 PMC7463519 32726920

[13] Xu M (2019). Genome-wide profiling of Epstein-Barr virus integration by targeted sequencing in Epstein-Barr virus associated malignancies. Theranostics.

[14] Cha YJ (2016). Clinicopathological and prognostic significance of programmed cell death ligand-1 expression in lung adenocarcinoma and its relationship with p53 status. Lung Cancer.

[15] Tojyo I (2019). PD-L1 expression correlated with p53 expression in oral squamous cell carcinoma. Maxillofac. Plast. Reconstr. Surg..

[16] Alexandrov LB (2013). Signatures of mutational processes in human cancer. Nature.

[17] Forbes SA (2010). COSMIC (the Catalogue of Somatic Mutations in Cancer): a resource to investigate acquired mutations in human cancer. Nucleic Acids Res..

[18] Lin DC (2014). The genomic landscape of nasopharyngeal carcinoma. Nat. Genet..

[19] Jiang L (2015). Positive expression of programmed death ligand-1 correlates with superior outcomes and might be a therapeutic target in primary pulmonary lymphoepithelioma-like carcinoma. Onco Targets Ther..

[20] Yu G, Wang LG, Han Y, He QY (2012). clusterProfiler: an R package for comparing biological themes among gene clusters. OMICS.

[21] Trapnell C (2010). Transcript assembly and quantification by RNA-Seq reveals unannotated transcripts and isoform switching during cell differentiation. Nat. Biotechnol..

[22] Yu X-Y (2018). Correlation and prognostic significance of PD-L1 and P53 expression in resected primary pulmonary lymphoepithelioma-like carcinoma. J. Thorac. Dis..

[23] VanderLaan PA (2017). Mutations in TP53, PIK3CA, PTEN and other genes in EGFR mutated lung cancers: correlation with clinical outcomes. Lung Cancer.

[24] McGowan M (2017). PIK3CA mutations as prognostic factor in squamous cell lung carcinoma. Lung Cancer.

[25] Singh, S. A. et al. PLZF targets developmental enhancers for activation during osteogenic differentiation of human mesenchymal stem cells. *Elife* **8**, e40364 (2019). 10.7554/eLife.40364 PMC6344081 30672466

[26] Zhang Q (2020). Expression of the PTEN/FOXO3a/PLZF signalling pathway in pancreatic cancer and its significance in tumourigenesis and progression. Investig. New Drugs.

[27] Stopsack KH (2019). Low expression of the androgen-induced tumor suppressor gene PLZF and lethal prostate cancer. Cancer Epidemiol. Biomark. Prev..

[28] Chen B (2018). Identification of fusion genes and characterization of transcriptome features in T-cell acute lymphoblastic leukemia. Proc. Natl Acad. Sci. USA.

[29] Shen H (2018). PLZF inhibits proliferation and metastasis of gallbladder cancer by regulating IFIT2. Cell Death Dis..

[30] Yousefnia S (2018). The influence of peroxisome proliferator-activated receptor γ (PPARγ) ligands on cancer cell tumorigenicity. Gene.

[31] Zhang Y (2016). Expression and function of PPARs in cancer stem cells. Curr. Stem Cell Res. Ther..

[32] Liu Y (2020). Telmisartan inhibits oxalate and calcium oxalate crystal-induced epithelial-mesenchymal transformation via PPAR-γ-AKT/STAT3/p38 MAPK-Snail pathway. Life Sci..

[33] Korpal M (2017). Evasion of immunosurveillance by genomic alterations of PPARγ/RXRα in bladder cancer. Nat. Commun..

[34] Seoane, J. & Gomis, R. R. TGF-β family signaling in tumor suppression and cancer progression. *Cold Spring Harb. Perspect. Biol*. **9**, a022277 (2017). 10.1101/cshperspect.a022277 PMC5710110 28246180

[35] Kariminik A, Kheirkhah B (2017). Tumor growth factor-β is an important factor for immunosuppression and tumorgenesis in Polyoma BK virus infection; a systematic review article. Cytokine.

[36] Mariathasan S (2018). TGFβ attenuates tumour response to PD-L1 blockade by contributing to exclusion of T cells. Nature.

[37] Xie Z (2020). A multicenter analysis of genomic profiles and PD-L1 expression of primary lymphoepithelioma-like carcinoma of the lung. Mod. Pathol..

[38] Zhang X (2017). Long noncoding RNA HNF1A-AS1 indicates a poor prognosis of colorectal cancer and promotes carcinogenesis via activation of the Wnt/β-catenin signaling pathway. Biomed. Pharmacother..

[39] Zhang J (2018). The oncogenic role of Epstein-Barr virus-encoded microRNAs in Epstein-Barr virus-associated gastric carcinoma. J. Cell. Mol. Med..

[40] Lung RW (2018). EBV-encoded miRNAs target ATM-mediated response in nasopharyngeal carcinoma. J. Pathol..

[41] Wang J (2019). Epstein-Barr virus miR-BART3-3p promotes tumorigenesis by regulating the senescence pathway in gastric cancer. J. Biol. Chem..

[42] Yoon, C. J. et al. Epstein-Barr virus-encoded miR-BART5-5p upregulates PD-L1 through PIAS3/pSTAT3 modulation, worsening clinical outcomes of PD-L1-positive gastric carcinomas. *Gastric Cancer* **23**, 780–795 (2020). 10.1007/s10120-020-01059-3 32206940

[43] Lyu X (2018). EBV-miR-BART1-5P activates AMPK/mTOR/HIF1 pathway via a PTEN independent manner to promote glycolysis and angiogenesis in nasopharyngeal carcinoma. PLoS Pathog..

[44] Gao W (2019). Detection of Epstein-Barr virus (EBV)-encoded microRNAs in plasma of patients with nasopharyngeal carcinoma. Head Neck.

[45] Zheng, X. et al. Epstein-Barr virus MicroRNA miR-BART5-3p inhibits p53 expression. *J. Virol*. **92**, e01022-18 (2018). 10.1128/JVI.01022-18 PMC6232473 30209170

[46] Chansky K (2017). The IASLC Lung Cancer Staging Project: external validation of the revision of the TNM stage groupings in the Eighth Edition of the TNM classification of lung cancer. J. Thorac. Oncol..

[47] Chen B (2017). Hyperphosphorylation of RPS6KB1, rather than overexpression, predicts worse prognosis in non-small cell lung cancer patients. PLoS ONE.

[48] Soria JC (2018). Osimertinib in untreated EGFR-mutated advanced non-small-cell lung cancer. N. Engl. J. Med..

[49] Lee H (2020). Analysis of tumor microenvironmental features to refine prognosis by T, N risk group in patients with stage III colon cancer (NCCTG N0147) (Alliance). Ann. Oncol..

[50] Dees ND (2012). MuSiC: identifying mutational significance in cancer genomes. Genome Res..

[51] Yang HJ (2013). Comprehensive profiling of Epstein-Barr virus-encoded miRNA species associated with specific latency types in tumor cells. Virol. J..

