# Supplementary material for: Molecular characteristics of primary pulmonary lymphoepithelioma-like carcinoma based on integrated genomic analyses
Source: Signal Transduct Target Ther. 2021 Jan 8;6:6. doi: 10.1038/s41392-020-00382-6 (PMC7791019; doi:10.1038/s41392-020-00382-6)
Supplement: Supplementary file 1 — Supplementary materials [file 41392_2020_382_MOESM1_ESM.docx]

Supplementary Materials for

Molecular Characteristics of Primary Pulmonary Lymphoepithelioma-like Carcinoma Based on Integrated Genomic Analyses

Bojiang Chen, Yu Zhang, Sisi Dai, Ping Zhou, Wenxin Luo, Zhoufeng Wang, Xuping Chen, Peng Cheng, Guoya Zheng, Jing Ren, Xiaodong Yang#, Weimin Li#

Correspondence to: [weimin003@163.com](mailto:weimin003@163.com); [yang-xiaodong@163.com](mailto:yang-xiaodong@163.com)

**This PDF file includes:**

Materials and Methods

Figures. S1 to S6

Tables S1 to S4

Captions for Data S1 to S2

**Other Supplementary Materials for this manuscript include the following:**

Data S1 to S2

Supplementary material 1, available online only;

Supplementary material 2, available online only.

Materials and Methods

IHC assay

All formalin-fixed paraffin-embedded (FFPE) samples were sectioned at 4 μm for the IHC assay. The workflow was carried out according to the manual of EnVision™ Detection Systems Peroxidase/DAB (Dako, Denmark, #K5007). Anti-PD-L1 (1:1200, 28-8, ab205921) and anti-p53 (1:100, E26, ab32389) were rabbit monoclonal antibodies from Abcam (Cambridge, UK). A heat-induced technique was used for epitope retrieval. After incubation with a biotinylated goat anti-rabbit antibody, the sections were incubated with peroxidase-conjugated streptavidin. 3,3’-Diamino-benzidine tetrahydrochloride (0.05%) was used as a chromogen. Phosphate-buffered saline (PBS) was used instead of the primary antibodies as a negative control.

The staining results were interpreted by two independent pathologists blinded to the clinicopathological data. According to previous reports, PD-L1 in tumor cells was evaluated as 0%, 1% - 49% and 50% or higher(1). Except for wild-type staining, P53 staining has 3 different mutation patterns: complete absence, overexpression and cytoplasmic. These mutation patterns are variably termed abnormal/aberrant/mutation-type and are strongly predictive of an underlying TP53 mutation. Complete absence is characterized by the presence of a positive internal control with staining of nonneoplastic cells such as lymphocytes, fibroblasts, or endothelial cells while overexpression is in the form of diffuse strong nuclear positivity involving at least 80% of the tumor cells but usually almost 100%. Other mutation-types and the normal/wild-type pattern with p53 expression levels are between these extreme. Wild-type staining is characterized by an admixture of negative cells, weakly and strongly positive cells and cytoplasmic pattern is characterized by an unequivocal cytoplasmic staining, which is accompanied by a variable nuclear staining (2).

WGS and RNA-seq

Two pathologists independently evaluated the tumor purity before sequencing, which varied from 20% - 80% and were satisfied that the samples met the sequencing requirements. Genomic DNA was extracted from fresh frozen tumor tissues and paracancerous tissues using a DNeasy Blood and Tissue Kit (Qiagen, Valencia, CA). For WGS sequencing, 200 ng genomic DNA was fragmented by sonication and constructed using library preparation with an NEBNext Ultra DNA Library Prep Kit (New England Biolabs, Ipswich, MA). The library was then sequenced using a high-throughput platform with a pair-end read of 150 bp using a HiSeq X sequencer (Illumina, San Diego, CA). Total RNA was extracted from the specimens using an RNeasy Mini Kit (Qiagen, Valencia, CA). A total amount of 2 μg rRNA-depleted RNA was used to prepare the sequencing libraries using an NEBNext Ultra RNA Library Prep Kit for Illumina (NEB, Ipswich, MA) following the manufacturer’s recommendations. Consequently, 125- to 150-bp paired-end reads were generated using an Illumina HiSeq platform. In addition, 6 μg of total RNA per sample was used as the input material for the small RNA library using the NEBNext Multiplex Small RNA Library Prep Set for Illumina (NEB, Ipswich, MA). Finally, 50-bp single-end sequencing was performed on an Illumina HiSeq 2500/2000 platform.

Genome alignment, host integration of EBV and variant calling

After removing sequencing reads with low quality and adapter bases, clean reads were aligned to the human genome (National Center for Biotechnology Information (NCBI) build 37, hg19) and EBV aggregated reference sequences using Burrows-Wheeler Aligner (BWA)-MEM. Sorted BAM files were created using SAMtools. The whole sample level for tumors and matched normal samples of each patient were produced by optical duplicate reads marked using Picard (<http://broadinstitute.github.io/picard/>). Single-nucleotide variants (SNVs) and small insertions and deletions (indels) were called and identified using Mutect2 and Strelka2.

Correlatively, EBV aggregated reference sequences contained 302 EBV genomes retrieved from NCBI GenBank; the aligned reads were assembled using SPAdes 3.13.0. The scaffolds were aligned to the EBV reference NC_007605.1, and the quality assessment was carried out using Quast. Facet was used to detect the CNV, while Crest was utilized for structural variant discovery. The somatic SNVs and indels were detected using muTect and Strelka, respectively. EBV integration-supportive signals were extracted from Crest.

References

1. Oyanagi J, Koh Y, Sato K, Mori K, Teraoka S, Akamatsu H, et al. Predictive value of serum protein levels in patients with advanced non-small cell lung cancer treated with nivolumab. Lung Cancer. 2019;132:107-13.

2. Köbel M, Ronnet B, Singh N, et al.Interpretation of P53 Immunohistochemistry in Endometrial Carcinomas: Toward Increased Reproducibility.International Journal of Gynecological Pathology 38:S123–S131.

Figure. S1.

**
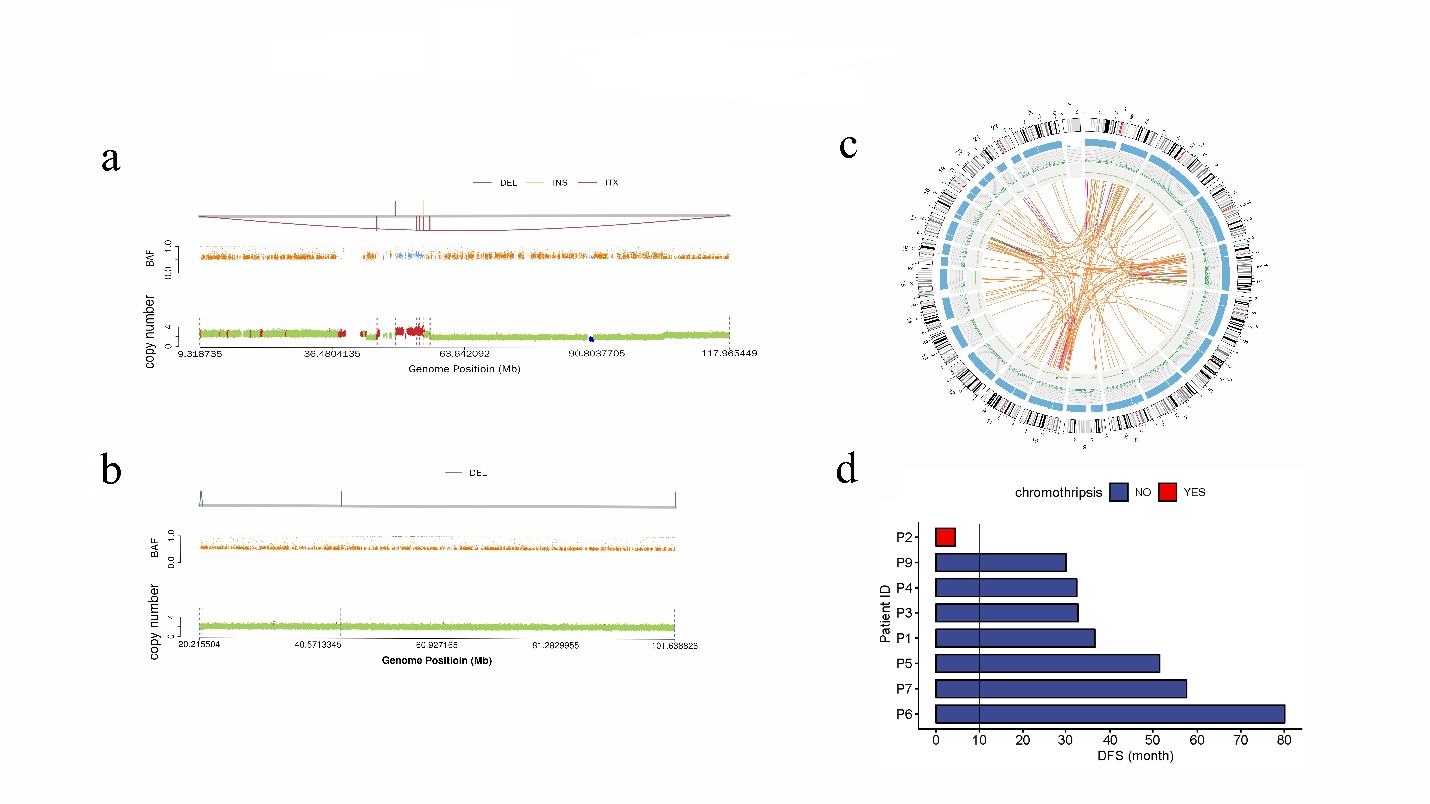
Chromothripsis analysis. (a)** Genome-wide copy number changes and B allele frequency (BAF) changes in a patient with chromothripsis. **(b)** Genome-wide copy number changes and BAF changes in a non-chromothripsis patient. **(c)** Massive rearrangement occurred in the chromothripsis sample. **(d)** Survival analysis of chromothripsis pLELC and non-chromothripsis pLELC.

Figure. S2.

**
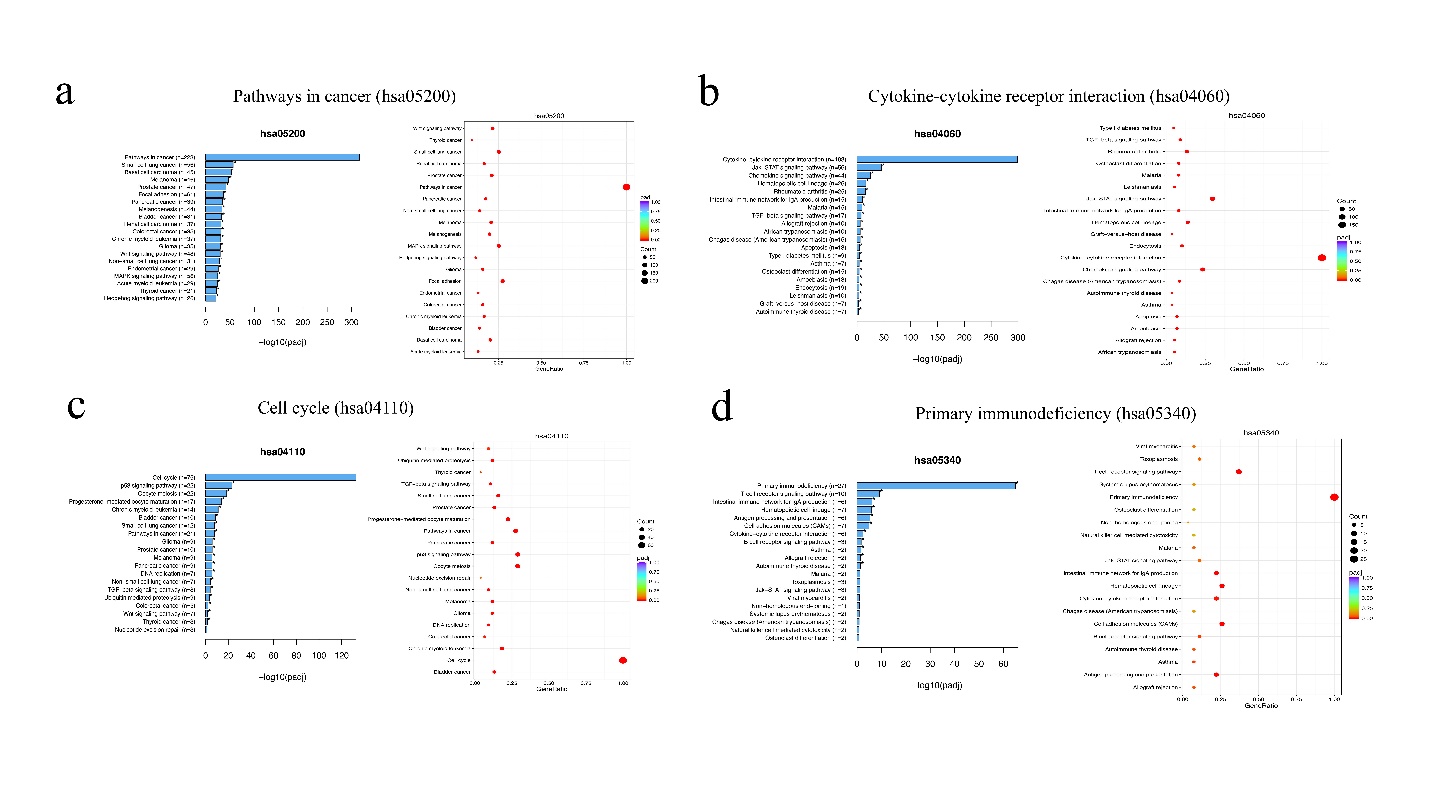
**

**Pathway enrichment analysis. (a)** Cancer, **(b)** cytokine-cytokine receptor interaction, **(c)** cell cycle, and **(d)** primary immunodeficiency.

Figure. S3.

**
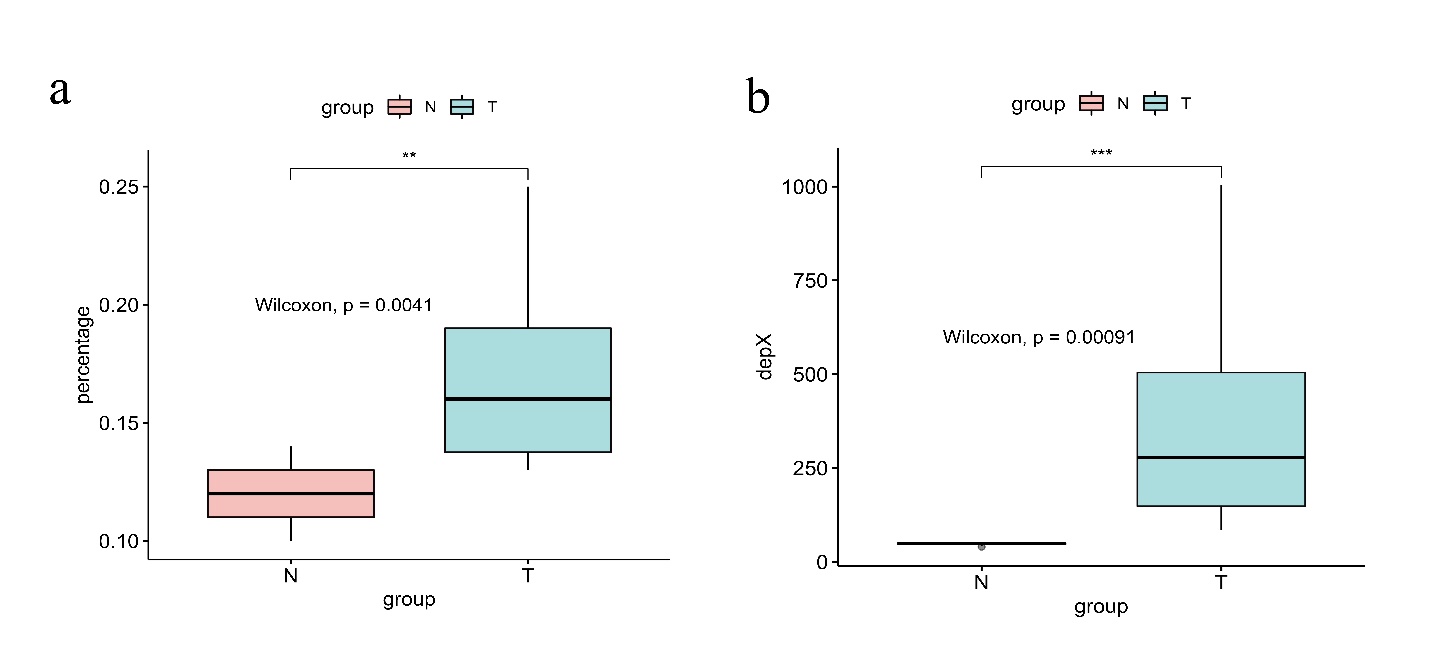
**

**The aligned integration comparison of WGS data of pLELC patients and EBV reference genomes.** N represents adjacent normal lung tissues and T stands for tumor tissue. **(a)** Percentage of reads mapped on EBV genome in two groups, *P* = 0.0041; **(b)** Average depth of reads mapped on EBV genome in two groups, *P* = 0.00091.

Figure. S4.

**
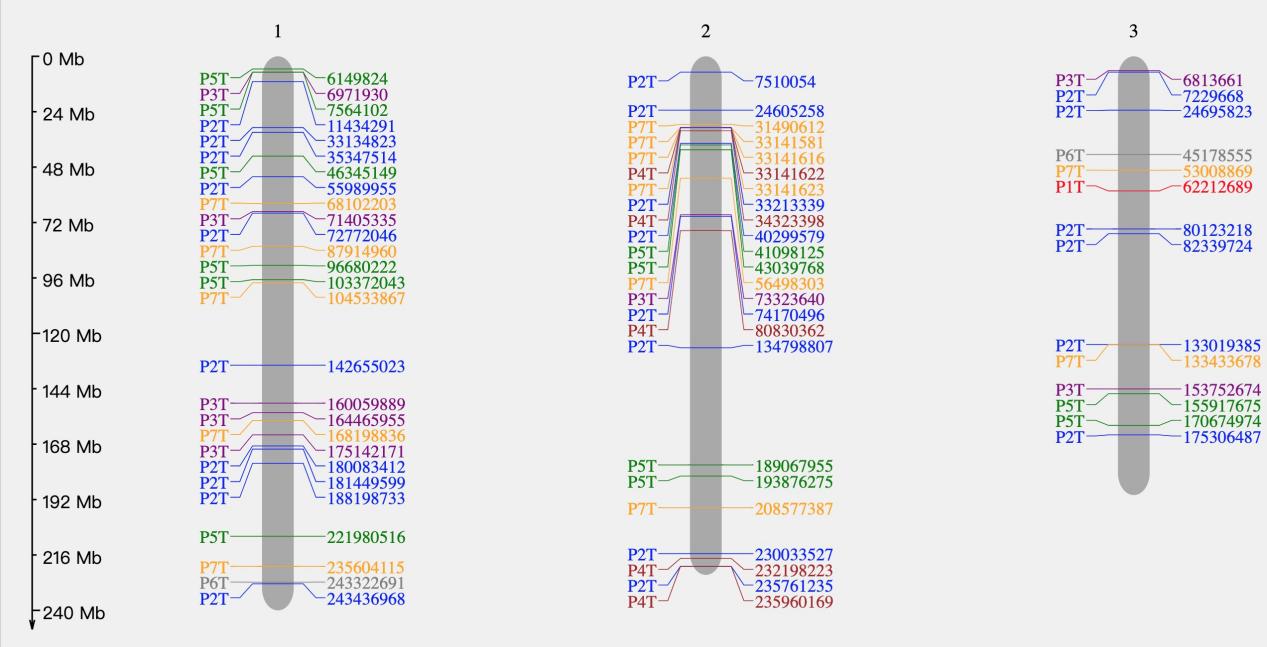
**

**
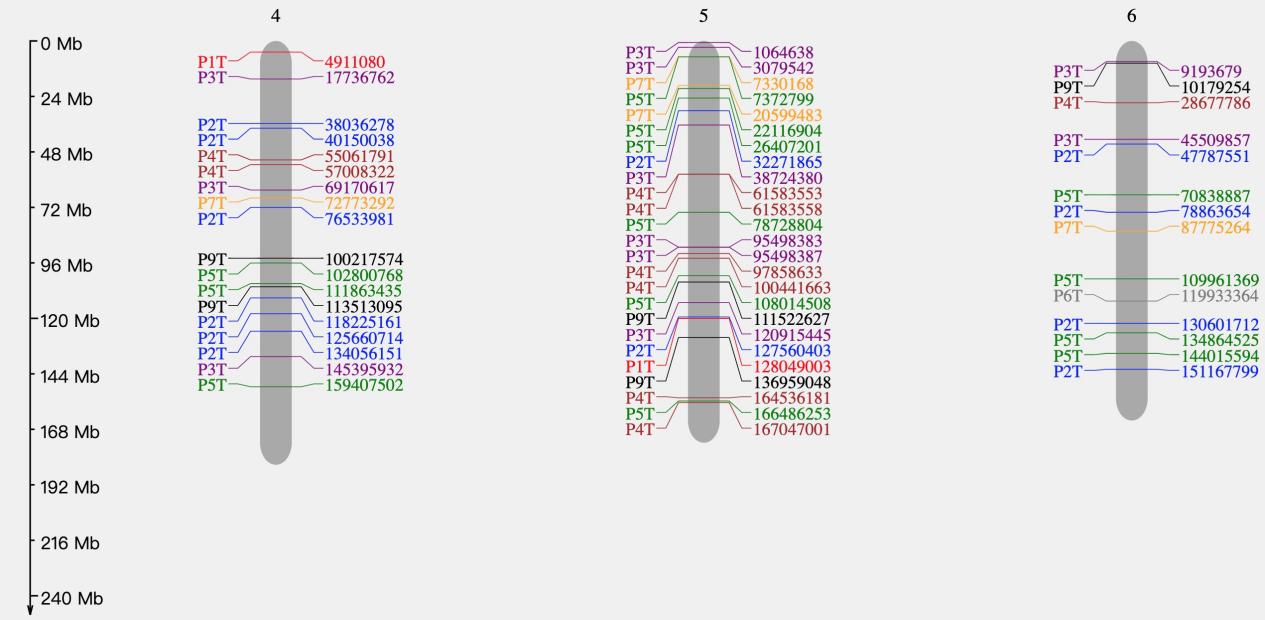
**

**
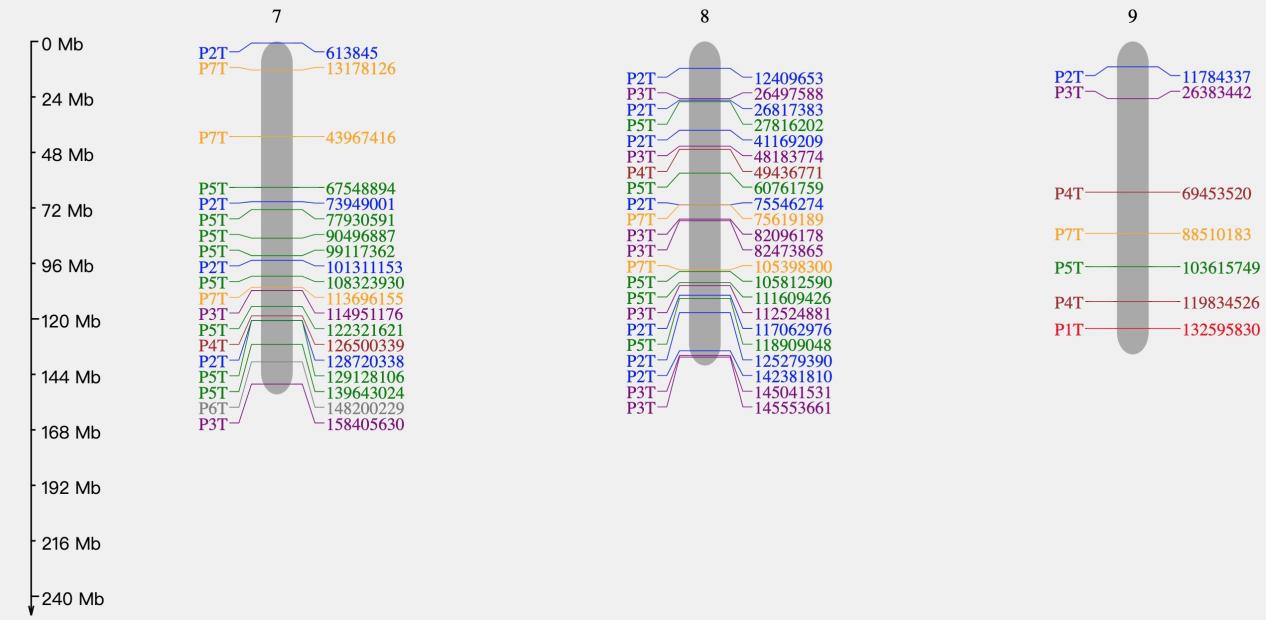
**

**
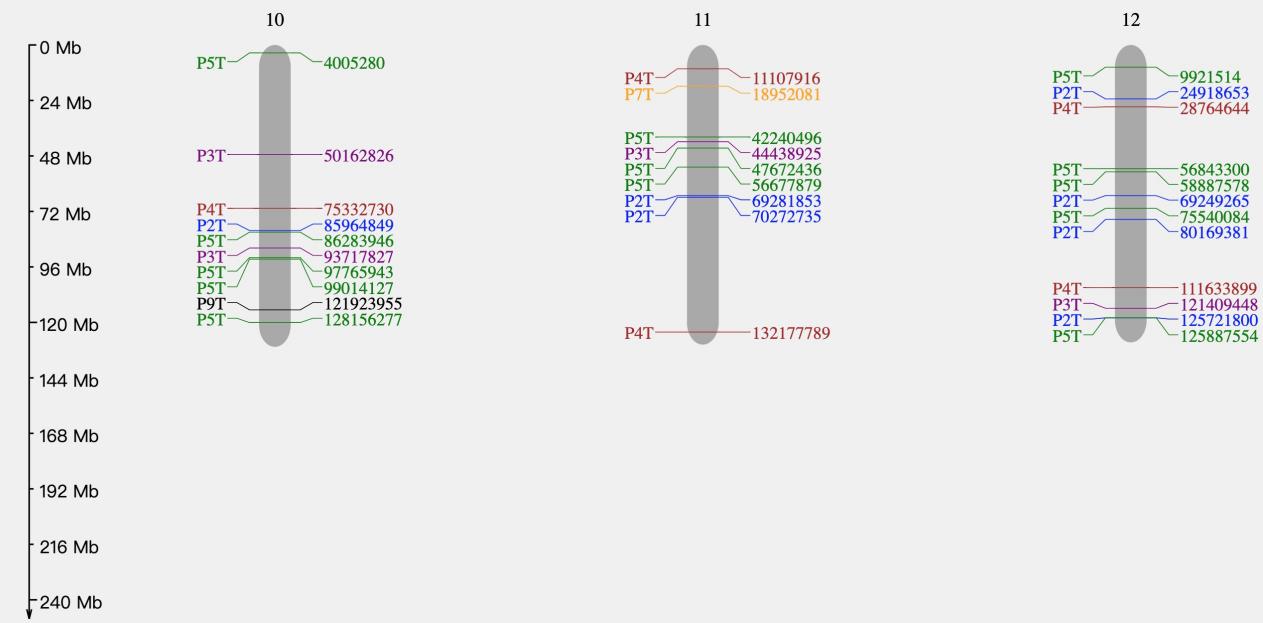
**

**
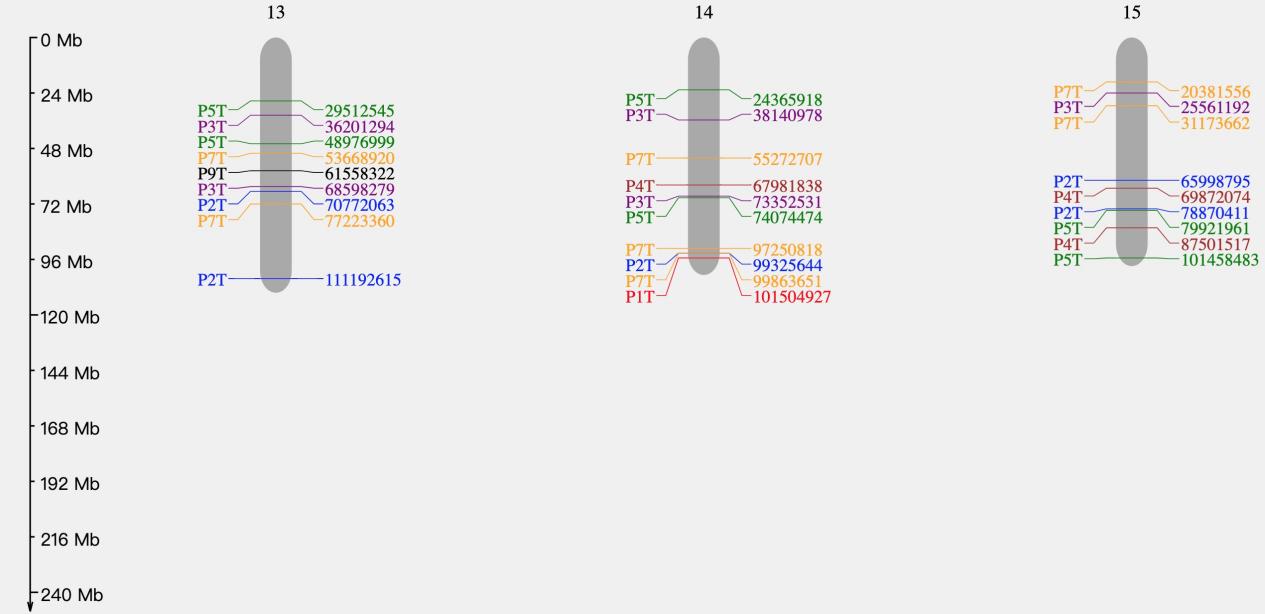
**

**
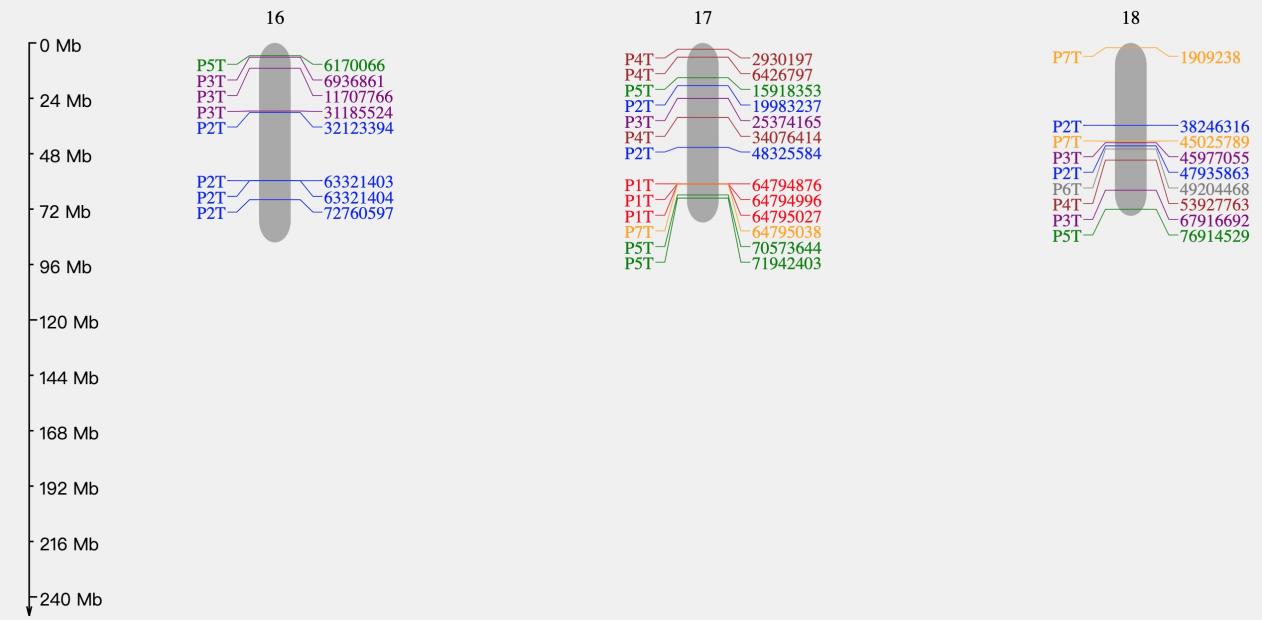
**

**
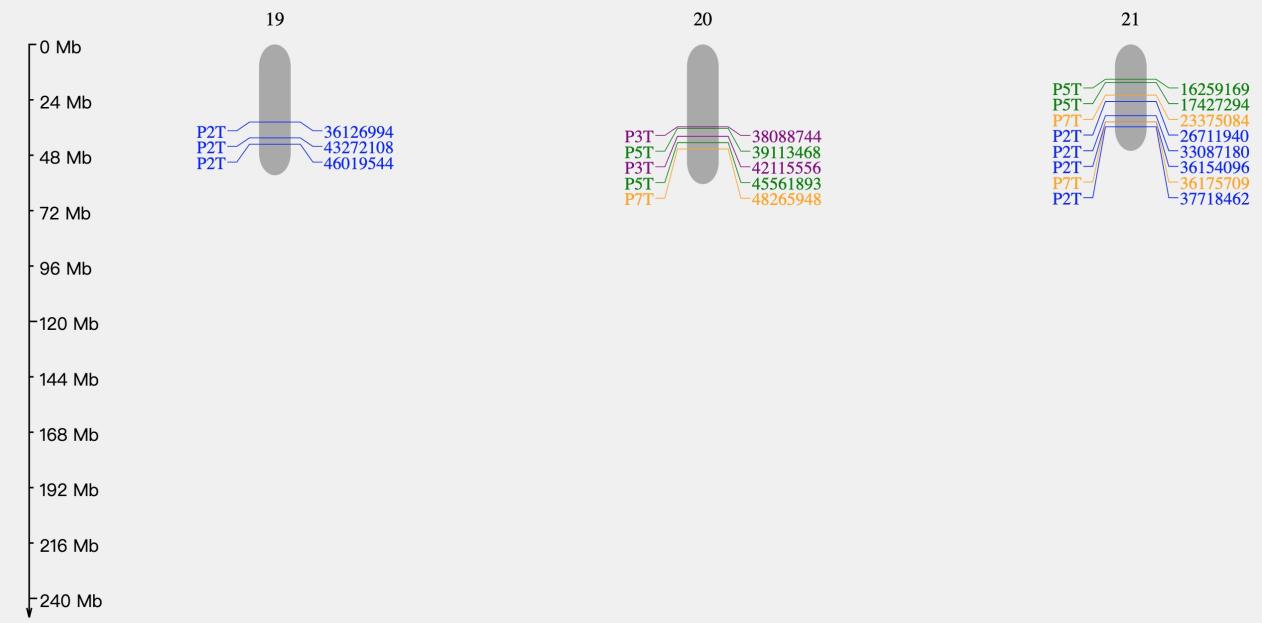
**

**
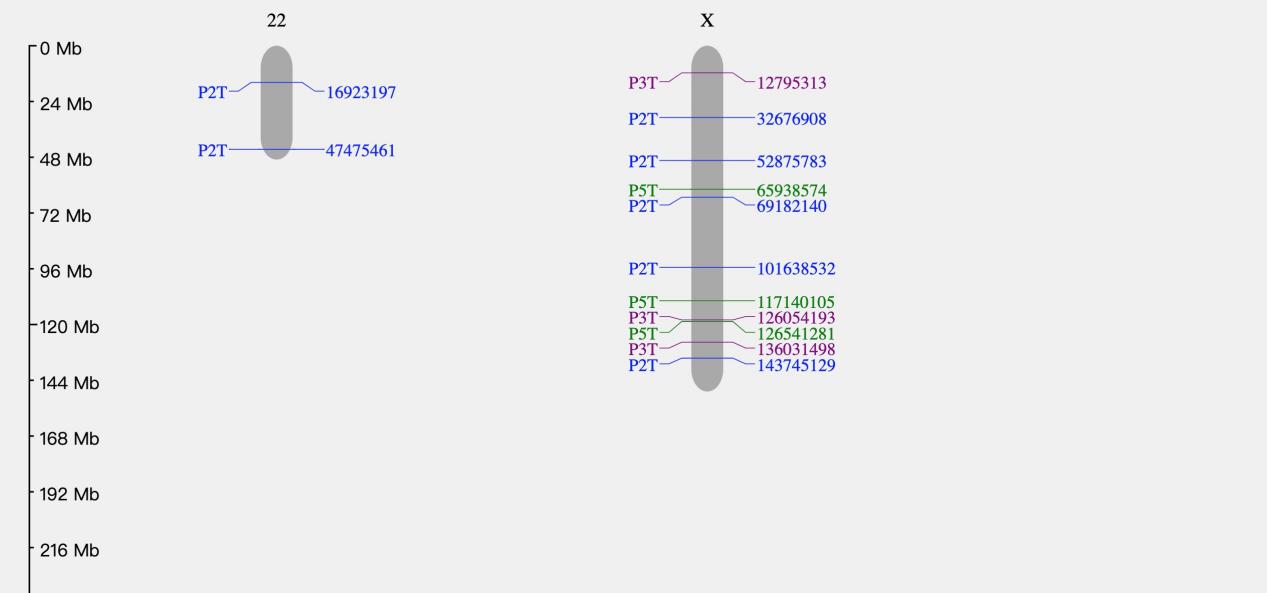
**

**The EBV integration breakpoint distribution in each patient.** Patients are numbered and represented by a range of colors. Chromosomes are displayed by the gray columns above which there is a number or ‘X’.

Figure. S5.

**
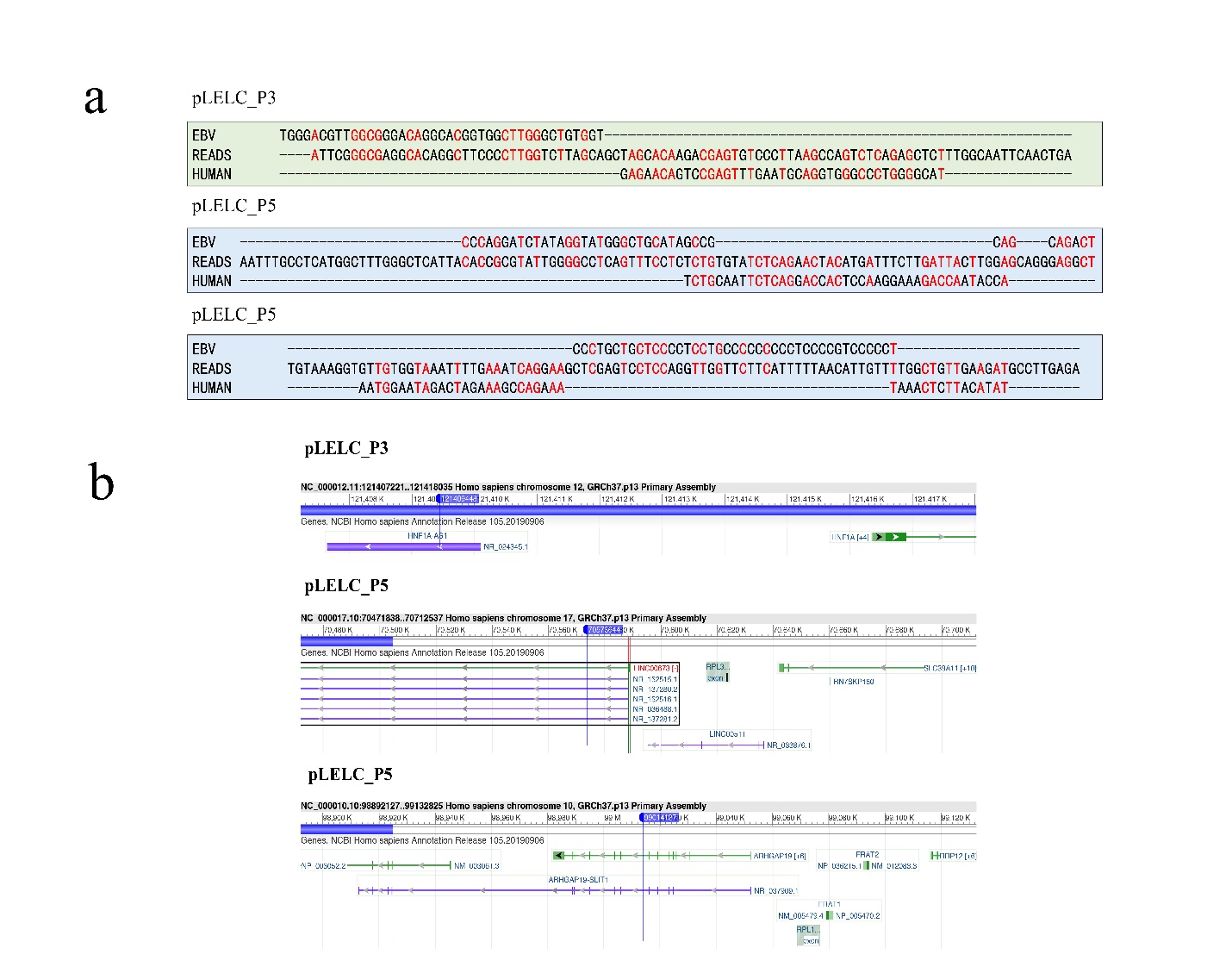
Integration sites of P3 and P5. (a)** Microhomology alignment between EBV, the human genome, and the integration sequences of P3 and P5. **(b)** EBV integration site in the human genome.

Figure. S6.

**
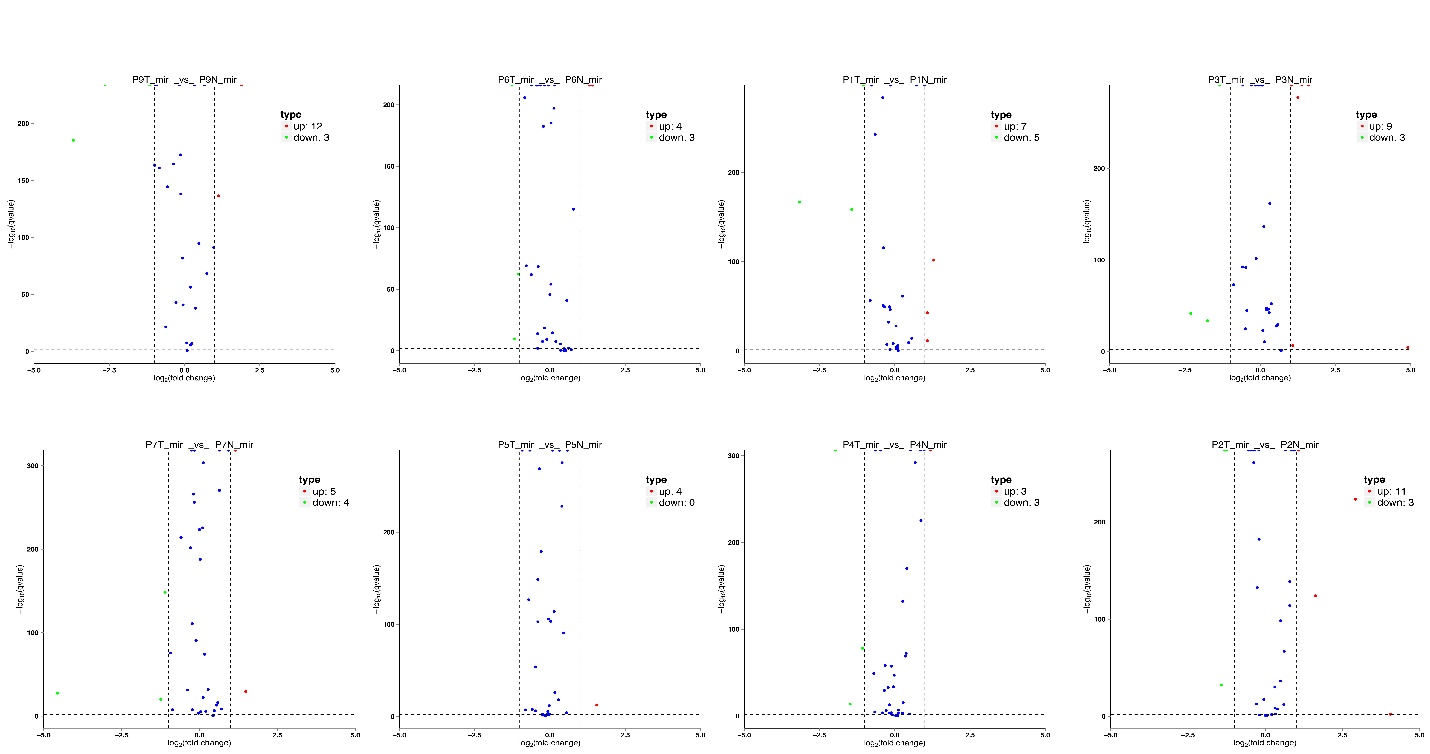
**

**Expression status of EBV-encoded miRNA in each patient.**

Table S1.

**Clinicopathological Characteristics of the pLELC patients for integrated genomic analyses (*n* = 8)**

| **Variables** | ***n* (%)** |
| --- | --- |
| **Age (Median, range, yrs)** | 51 (44-59) |
| **Gender** |  |
| Male | 1 (12.5) |
| Female | 7 (87.5) |
| **Smoking** |  |
| No | 8 (100) |
| **Family history of cancer** |  |
| No | 8 (100) |
| **Clinical stage** |  |
| Ⅰ | 3 (37.5) |
| Ⅱ | 3 (37.5) |
| Ⅲ | 2 (25.0) |
| **EREB status** |  |
| Positive | 8 (100) |

Table S2.

**Eight common pairs of EBV integration site**

| P5T X 126541281 . intergenic  NM_001122716,NM_138289 |
| --- |
| P3T X 126054193 . intergenic  NM_001122716,NM_138289 |
| P3T 8 112524881 . intergenic  NM_014379,NM_198123 |
| P5T 8 111609426 . intergenic  NM_014379,NM_198123 |
| P7T 8 75619189  FLJ39080  ncRNA_intronic  NR_033830 |
| P2T 8 75546274  FLJ39080  ncRNA_intronic  NR_033830 |
| P7T 17  64795038  PRKCA intronic  NM_002737 |
| P1T 17  64795027  PRKCA intronic  NM_002737 |
| P1T 17  64794996  PRKCA intronic  NM_002737 |
| P1T 17  64794876  PRKCA intronic  NM_002737 |
| P7T 2 33141623  LINC00486 ncRNA_intronic  NR_027098,NR_027099,NR_027100 |
| P4T 2 33141622  LINC00486 ncRNA_intronic  NR_027098,NR_027099,NR_027100 |
| P7T 2 33141616  LINC00486 ncRNA_intronic  NR_027098,NR_027099,NR_027100 |
| P7T 2 33141581  LINC00486 ncRNA_intronic  NR_027098,NR_027099,NR_027100 |
| P9T 6 10179254  . intergenic  NR_038980,NM_001042425 |
| P3T 6 9193679 . intergenic  NR_038980,NM_001042425 |
| P5T 1 7564102 CAMTA1  intronic  NM_015215 |
| P3T 1 6971930 CAMTA1  intronic  NM_015215 |
| P5T 5 7372799 . intergenic  NR_033906,NM_020546 |
| P7T 5 7330168 . intergenic  NR_033906,NM_020546 |

*Screening criteria: at least 2 integration sites from different samples are located in the same intragenic or intergene region

Table S3.

**Percentage of EBV-miRNAs found in small-RNA sequencing of EBV-associated pLELCs**

| **Patients** | **EBV mapped** | **EBV mapped positive (%)** | **% of EBV miRNA** |
| --- | --- | --- | --- |
| P1 | 2920385 | 2363907 (80.9) | 27.3 |
| P2 | 1672080 | 1362103 (81.5) | 27.3 |
| P3 | 3557075 | 2985904 (83.9) | 25.0 |
| P4 | 2697913 | 2163709 (80.2) | 13.6 |
| P5 | 4050387 | 3451712 (85.2) | 9.1 |
| P6 | 430873 | 368356 (85.5) | 15.9 |
| P7 | 762824 | 643925 (84.4) | 20.5 |
| P9 | 1602705 | 1408493 (80.9) | 34.1 |

Note: P8 was excluded from the integrated genomic analyses due to the metastatic nasopharyngeal carcinoma.

Table S4

**Expression and functional prediction of miR-BARTs in EBV.**

|  | **Patients detected (*n*)** | **Patients with high expressions (*n*)** | **Target genes prediction** |
| --- | --- | --- | --- |
| **BART5-3p** | **8** | **7** | **TP53 [27]** |
| **BART20-3p** | **8** | **7** | **/** |
| BART10-5p | 8 | 5 | / |
| BART20-5p | 8 | 4 | Bcl-2 [28]; T-bet [29] |
| BART11-3p | 8 | 3 | / |
| BART10-3p | 8 | 2 | DKK1 [30] |
| BART2-3p | 8 | 2 | / |
| BART3-3p | 8 | 2 | TP53 [54] |
| BART12 | 8 | 1 | / |
| BART13-3p | 8 | 1 | / |
| BART13-5p | 8 | 1 | / |
| BART16 | 8 | 1 | LMP1 [33] |
| BART17-5p | 8 | 1 | / |
| BART18-3p | 8 | 1 | / |
| BART19-5p | 8 | 1 | / |
| BART21-3p | 8 | 1 | / |
| BART2-5p | 8 | 1 | / |
| BART4-3p | 8 | 1 | / |
| BART5-5p | 8 | 1 | ATM [25] |
| BART7-5p | 8 | 1 | / |
| BART9-5p | 8 | 1 | / |

Data S1. (separate file)

Supplementary material 1, available online only

Data S2. (separate file)

Supplementary material 2, available online only
